# A novel *AST2* mutation generated upon whole-genome transformation of *Saccharomyces cerevisiae* confers high tolerance to 5-Hydroxymethylfurfural (HMF) and other inhibitors

**DOI:** 10.1371/journal.pgen.1009826

**Published:** 2021-10-08

**Authors:** Gert Vanmarcke, Quinten Deparis, Ward Vanthienen, Arne Peetermans, Maria R. Foulquié-Moreno, Johan M. Thevelein

**Affiliations:** 1 Laboratory of Molecular Cell Biology, Institute of Botany and Microbiology, KU Leuven, Leuven-Heverlee, Belgium; 2 Center for Microbiology, VIB, Leuven-Heverlee, Belgium; 3 NovelYeast bv, Open Bio-Incubator, Erasmus High School, Brussels (Jette), Belgium; University of Rochester, UNITED STATES

## Abstract

Development of cell factories for conversion of lignocellulosic biomass hydrolysates into biofuels or bio-based chemicals faces major challenges, including the presence of inhibitory chemicals derived from biomass hydrolysis or pretreatment. Extensive screening of 2526 *Saccharomyces cerevisiae* strains and 17 non-conventional yeast species identified a *Candida glabrata* strain as the most 5-hydroxymethylfurfural (HMF) tolerant. Whole-genome (WG) transformation of the second-generation industrial *S*. *cerevisiae* strain MD4 with genomic DNA from *C*. *glabrata*, but not from non-tolerant strains, allowed selection of stable transformants in the presence of HMF. Transformant GVM0 showed the highest HMF tolerance for growth on plates and in small-scale fermentations. Comparison of the WG sequence of MD4 and GVM1, a diploid segregant of GVM0 with similarly high HMF tolerance, surprisingly revealed only nine non-synonymous SNPs, of which none were present in the *C*. *glabrata* genome. Reciprocal hemizygosity analysis in diploid strain GVM1 revealed *AST2*^N406I^ as the only causative mutation. This novel SNP improved tolerance to HMF, furfural and other inhibitors, when introduced in different yeast genetic backgrounds and both in synthetic media and lignocellulose hydrolysates. It stimulated disappearance of HMF and furfural from the medium and enhanced *in vitro* furfural NADH-dependent reducing activity. The corresponding mutation present in *AST1* (i.e. *AST1*^D405I^) the paralog gene of *AST2*, also improved inhibitor tolerance but only in combination with *AST2*^N406I^ and in presence of high inhibitor concentrations. Our work provides a powerful genetic tool to improve yeast inhibitor tolerance in lignocellulosic biomass hydrolysates and other inhibitor-rich industrial media, and it has revealed for the first time a clear function for Ast2 and Ast1 in inhibitor tolerance.

## Author summary

The use of lignocellulosic biomass from waste streams or energy crops is highly favored over the use of fossil resources for the production of biofuels or bio-based chemicals in the fight against climate change. However, the pretreatment and hydrolysis of the biomass generates large amounts of inhibitors that compromise the subsequent fermentation of the released sugars. In this work, we have used a rarely applied technology, whole-genome transformation with DNA from an inhibitor tolerant species to obtain cellulosic yeast strains with improved inhibitor tolerance. This resulted in a new highly efficient gene tool, *AST2*^N406I^, for targeted improvement of inhibitor tolerance in different yeast strain backgrounds and active against multiple inhibitors. A highly surprising result from this work is that the origin of the donor DNA from an inhibitor tolerant strain is essential to obtain stable inhibitor-tolerant transformants by whole-genome transformation but that none of the mutations, including the causative mutation, *AST2*^N406I^, was present in the genomic DNA of the donor strain. A tentative explanation is that incoming protective DNA fragments are maintained as extrachromosomal DNA, allowing proliferation of the host strain under the selective condition until it can generate itself a spontaneous mutation in its own DNA that takes over the protective function, after which the heterologous DNA is easily lost.

## Introduction

Second-generation bioethanol, produced from lignocellulosic biomass hydrolysates, is a promising alternative transport fuel with multiple major benefits over fossil fuels and first-generation bioethanol. However, for cost-efficient production of second-generation (2G) bioethanol several hurdles have to be overcome. With respect to the industrial yeast strains used for fermentation, the lack of xylose utilization has been successfully addressed by engineering heterologous genes from xylose-utilizing organisms, for instance the bacterial xylose isomerase from *Clostridium phytofermentans* [1]. A second challenge is the high level of inhibitors present in lignocellulose hydrolysates that severely reduce the yeast fermentation rate and yield, in particular that of xylose [2,3]. In general, the cheaper, harsh methodologies used for pretreatment of the lignocellulosic biomass result in higher levels of inhibitors, while the more gentle methodologies that result in less toxicity compromise the economic viability of the industrial process due to their higher cost [4–6]. The higher temperature used in processes like simultaneous saccharification and fermentation, and consolidated bioprocessing, further increases the toxicities of ethanol and the inhibitory compounds in lignocellulosic hydrolysates [7]. In addition to the production of bioethanol from lignocellulosic biomass, these challenges also apply to the production of other bio-based chemicals with 2G yeast cell factories, in which toxicity of many of these chemicals further aggravates the burden for the yeast.

Inhibitors identified in lignocellulose hydrolysates include acetic acid, 5-hydroxymethylfurfural (HMF), furfural, formic acid, levulinic acid, vanillin, 4-hydroxybenzaldehyde and 4-hydroxybenzoic acid [8]. Although inhibitor profiles of lignocellulose hydrolysates vary greatly depending on the biomass origin and the type of pretreatment, the furan aldehydes, HMF and furfural, as well as acetic acid, always seem to be among the most toxic inhibitors. The aldehyde group in furan aldehydes affects DNA, RNA, proteins and membranes, and causes accumulation of reactive oxygen species [9,10]. Moreover, HMF inhibits activity of multiple enzymes, affects lag phase length and induces apoptosis [11]. NAD(P)H-dependent conversion of the furan aldehyde group into the lesser toxic alcohol group in 2,5-bis-hydroxymethylfuran or in 2-furanmethanol, results in in-situ detoxification of HMF and furfural, respectively. Hence, higher conversion capacity generally supports higher tolerance to HMF and furfural. For HMF, genes known to encode enzymes that catalyze this reaction are *ADH1*, *ADH6*, *ADH7*, *ALD6*, *ARI1* and *GRE2*. For furfural, these include in addition *ALD4*, *YDR541C*, *YGL039W*, *YNL134C* and the recently identified *YKL107W* [12–21]. All these genes have been identified by conferring higher tolerance upon overexpression. Similar aldehyde detoxification mechanisms have been described in other yeast species [22]. Few other detoxification methods have been reported. Conversion of furfural into formic acid under aerobic conditions, and overexpression of a mutant allele of the general stress response gene *YAP1* or of the cofactor regeneration gene *ZWF1*, have been described [23–25]. Also, treatment of the medium with recombinant manganese peroxidase reduces furan aldehyde levels [26]. Only few specific mutations, *ADH1*^S110P^, *ADH1*^Y295C^ and *YAP1*^C620F^, have been linked to improved furfural and HMF tolerance [23,27]. Hence, it is of interest to identify novel, stable mutations that can be engineered in 2G industrial strains to improve their HMF and furfural tolerance.

Several genetic modifications have been described that enhance acetic acid tolerance, of which overexpression of the Haa1 transcription factor has been documented in multiple reports [28–30]. Improvement of inhibitor tolerance by evolutionary adaptation in media with increasing inhibitor levels has been described for HMF, furfural and acetic acid [31,32]. Stability of the strains obtained with evolutionary adaptation is often a major issue. In addition, these strains often suffer from unexpected side-effects due to detrimental mutations generated in the background of the strain. Hence, it is important to identify the causative genetic elements responsible for the improved inhibitor tolerance and introduce only these specific genetic modifications in 2G industrial yeast strains.

Whole-genome transformation (WGT) has been described as a method to transfer genetic factors responsible for a trait of interest from a donor strain to a recipient strain. In WGT, the complete genomic DNA (gDNA) of a strain superior for a trait of interest is transformed into a recipient host strain, after which the superior transformants are isolated under selective conditions for the trait. This has been applied with bacterial strains of the same species, for transfer of traits between *S*. *cerevisiae* strains and for introduction of traits from other yeast species into *S*. *cerevisiae* [9,33–37]. Very little information is available on the genetic basis of improved traits in WG *S*. *cerevisiae* transformants. Surprisingly, WGT has not been used for improvement of industrially relevant traits or for identification of genetic factors underlying industrially important traits, except for our recent report in which we used it as the donor for WGT to improve acetic acid tolerance in an industrial 2G yeast strain [38]. In addition, we have recently reported on the isolation of WG transformants with improved thermotolerance using gDNA from thermotolerant *Kluyveromyces marxianus* and *Ogataea polymorpha* strains. This surprisingly revealed that the transformants did not contain any gDNA sequences from the donor strains and that the very few SNPs present, including the causative SNPs identified in each transformant, also did not originate from the donor strain. In spite of this, transformation with gDNA from a thermotolerant donor strain was essential to obtain stable thermotolerant transformants. We suggested that transient presence of donor DNA in the host facilitates proliferation at high temperature and thus increases the chances for occurrence of spontaneous mutations suppressing the poor growth at high temperature [39].

In this work, we have used WGT for improvement of HMF tolerance in an industrial 2G *S*. *cerevisiae* strain. First, a *Candida glabrata* strain was identified as the most HMF tolerant in our yeast strain collection. Next, we used the gDNA of this *C*. *glabrata* strain as donor for WGT of the *S*. *cerevisiae* strain and obtained multiple transformants with improved HMF tolerance. In GVM0, the most HMF tolerant transformant, very few SNPs were introduced after WGT, yet none of these SNPs were originating from the *C*. *glabrata* DNA. A mutation in *AST2* was identified as the sole causative genetic modification introduced in GVM0 via WGT. Expression of the mutant *AST2* allele in multiple industrial yeast strain backgrounds improved tolerance not only to HMF but also to furfural, as well as other inhibitors like vanillin and acetic acid, during fermentation in inhibitor-spiked lignocellulosic biomass hydrolysate. Our work provides a new powerful genetic tool for improvement of yeast tolerance against various inhibitors present in lignocellulose hydrolysates and other inhibitor-rich industrial media.

## Results

### Screening of yeast strain collection for high HMF tolerance

**Fig 1:**
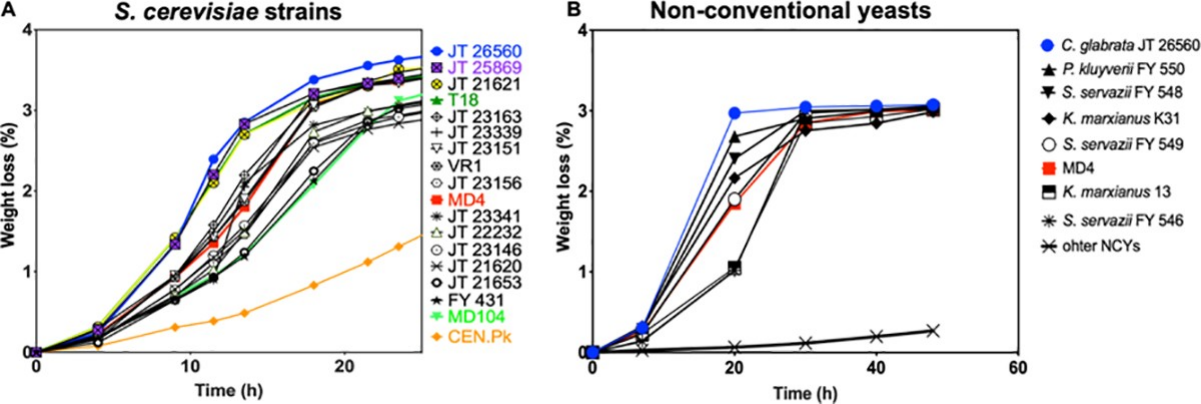
Fermentation performance of the yeast strains with highest HMF tolerance and control strains in small-scale semi-anaerobic fermentations in the presence of a high HMF concentration. A. *S*. *cerevisiae* strains and *C*. *glabrata*. B. Non-conventional yeast species and *S*. *cerevisiae* MD4 control strain. Semi-anaerobic small-scale fermentations (10 mL) with the yeast strains identified as most tolerant to HMF for growth on solid nutrient plates, in YPD6.5% with 8 g/L HMF, pH 5.2, 35°C and initial OD 5.0. Representative result of two biological replicates is shown.

In this work, we have focused on improvement of HMF tolerance. For that purpose, we first evaluated HMF tolerance of 2526 *S*. *cerevisiae* strains, as well as 17 non-conventional yeast species previously reported as displaying high HMF tolerance (Mukherjee, 2016), during growth on solid nutrient medium with a high HMF concentration (8 g/L). We identified 17 *S*. *cerevisiae* strains and four non-conventional yeast species with superior HMF tolerance under this condition. These strains, as well as three industrial 2G *S*. *cerevisiae* strains, MD4, T18 and MD104, and the lab strain CEN.PK as controls, were subsequently evaluated for HMF tolerance (8 g/L) in small-scale semi-anaerobic fermentations with synthetic medium (**Fig 1**). This resulted in the identification of a *Candida glabrata* strain (JT26560) as the most HMF tolerant strain of all strains evaluated (**Fig 1**). The 2G industrial *S*. *cerevisiae* strains, MD4, T18 and MD104, as well as three non-conventional yeast species, *P*. *kluyveri*, *K*. *marxianus* and *S*. *servazii* also displayed superior fermentation performance in presence of a high HMF concentration. All other *S*. *cerevisiae* strains, including the lab strain CEN.PK, as well as all other non-conventional yeast species tested clearly showed a much poorer fermentation performance under these conditions.

### Isolation of HMF-tolerant transformants of MD4 using WGT with *C*. *glabrata* gDNA

**Fig 2:**
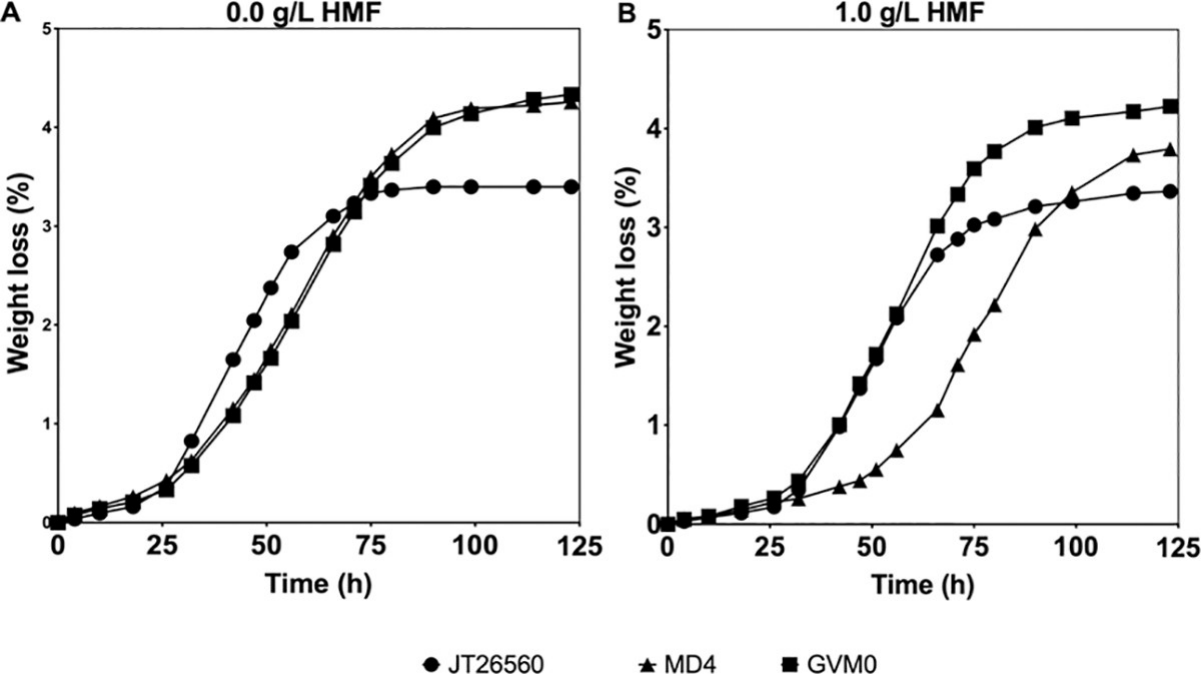
Fermentation performance of WG transformant GVM0 in HMF-enriched corn cob hydrolysate. Small-scale fermentations (10 mL) were performed in corn cob hydrolysate enriched with 0.0 g/L or 1.0 g/L HMF, pH 5.2, 35°C, 350 rpm and initial OD 5.0. (JT2650, *C*. *glabrata*; GVM0, WGT of MD4). Representative result of two biological replicates is shown.

We have performed WGT of the 2G industrial yeast strain MD4 using gDNA from the most HMF-tolerant strain, *C*. *glabrata* JT26560, four HMF-tolerant *S*. *cerevisiae* strains, the three other non-conventional yeast species with highest HMF tolerance, *P*. *kluyveri*, *K*. *marxianus* and *S*. *servazii*, five non-HMF-tolerant *S*. *cerevisiae* strains, as well as the recipient host strain MD4 itself and just water as another control. The transformants were selected on solid YPDX plates with 2.5 g/L HMF. After restreaking on solid nutrient medium with 2.5 g/L or 4.0 g/L HMF, and stability analysis by re-culturing in YPDX and freeze/thawing, we noticed that only in case of WGT with gDNA of the HMF tolerant *C*. *glabrata* strain, we were able to obtain stable MD4 transformants displaying improved HMF tolerance. The transformant strain GVM0 displayed the highest HMF tolerance of all transformants in small-scale semi-anaerobic fermentations with YPDX medium and 1.0 g/L HMF (**S1 Fig**). Fermentations in HMF-enriched corn cob hydrolysate revealed that the GVM0 transformant clearly showed improved HMF tolerance compared to the recipient host strain MD4 and the gDNA donor *C*. *glabrata* strain (**Fig 2**). In the absence of spiked HMF, GVM0 and MD4 displayed the same fermentation performance in corn cob hydrolysate, indicating absence of negative-side effects from the WGT procedure, at least under this condition (**Fig 2**). The ethanol titer after 72 h was reduced with 51% for MD4 in corn cob hydrolysate spiked with 1.0 g/L HMF, whereas for GVM0 it was reduced with only 4%.

### Identification of the causative genetic modification in strain GVM1 for enhanced HMF tolerance

The tetraploid WG transformant, GVM0, was sporulated and a diploid segregant, GVM1, was isolated that showed similar fermentation performance in corn cob hydrolysate spiked with HMF as compared to the parent strain GVM0 (**S2 Fig**). Subsequently, whole-genome sequencing and bio-informatics analysis of GVM1 and MD4 revealed only nine non-synonymous single nucleotide polymorphisms (SNPs) between both strains (Table 1), as well as multiple synonymous SNPs. All non-synonymous SNPs were absent in the genome of the *C*. *glabrata* gDNA donor strain used for WGT and were present in heterozygous form in strain GVM1. The nine non-synonymous SNPs have not previously been linked to furan aldehyde tolerance and had a variable presence in the genome of 1011 WG sequenced *S*. *cerevisiae* strains (Peter et al., 2018).

**Table 1:** List of the nine non-synonymous SNPs present in GVM1, segregant of the WG transformant GVM0, compared to the parent strain MD4. SNP frequency was determined as percentage of 1011 whole-genome sequenced strains that contained the non-synonymous SNP.

Gene	Chromosome	Mutation	Non-synonymous SNP	SNP frequency in *S*. *cerevisiae* strains
*REG2*	II	C to A	A239S	3.1%
*SAS3*	II	CG to TC	R625S	1.4%
*DPP1*	IV	A to G	I246T	26.7%
*GIC2*	IV	T to C	I6V	5.5%
*AST2*	V	T to A	N406I	1.0%
*IES1*	VI	A to G	L589P	0.0%
*ASG1*	IX	C to T	S899P	12.0%
*SYC1*	XV	A to C	N18K	1.3%
*TAH18*	XVI	A to C	T214N	18.8%

**Fig 3:**
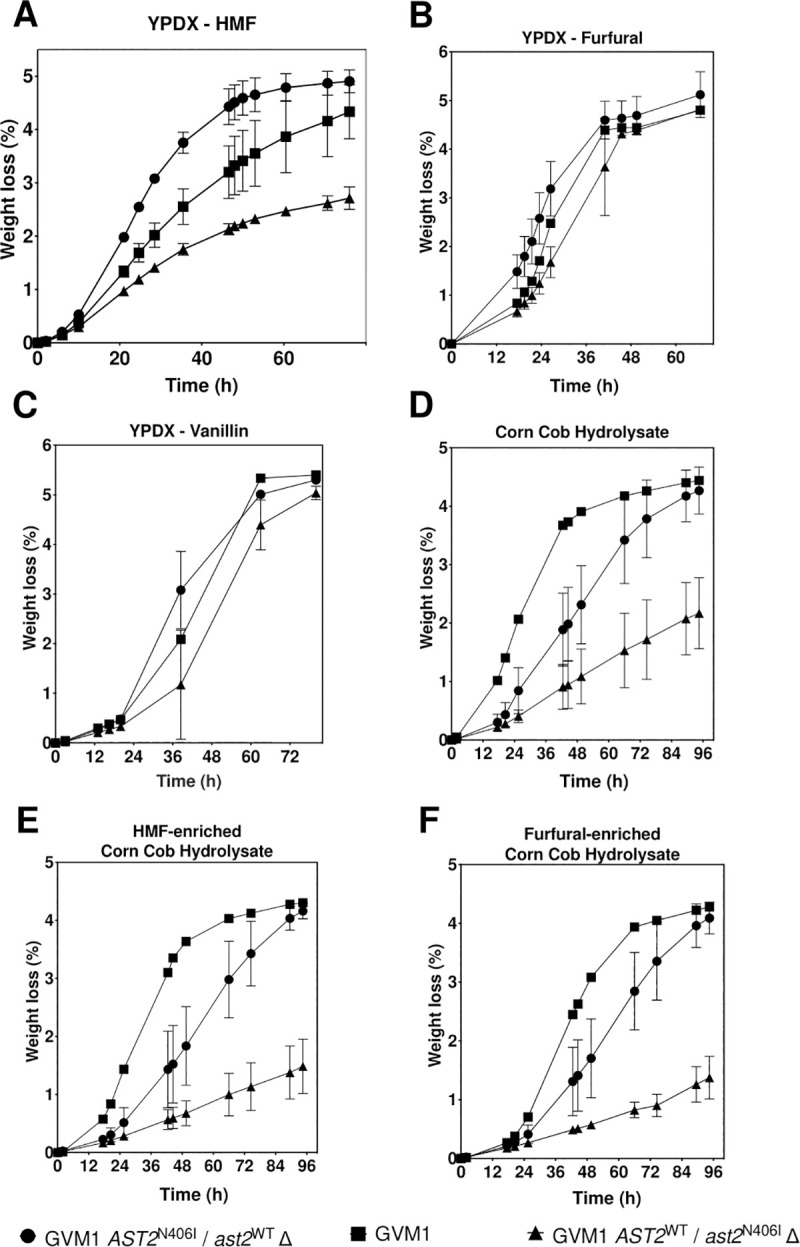
Fermentation performance of the two hemizygous strains of WG transformant GVM1 containing either the mutant *AST2*^N406I^ allele or the wild-type *AST2* allele. Small-scale fermentations (10 mL) were performed at pH 5.2, 35°C, 350 rpm and initial OD 5.0, in YPDX medium enriched with 12.0 g/L HMF (A), 4.0 g/L furfural (B), or 4.5 g/L vanillin (C), and in corn cob hydrolysate with no addition (D), or enriched with 1.0 g/L HMF (E) or 1.0 g/L furfural (F). Mean values with standard deviation are shown for four independent transformants of strain GVM1, or four technical replicates for strain GVM1. Representative result of two biological replicates is shown.

Next, reciprocal hemizygosity analysis (RHA) was performed to identify which SNP(s) were responsible for the enhanced HMF tolerance in strain GVM1. For that purpose, for each of the nine genes with a non-synonymous SNP introduced by WGT, the mutant or the wild type allele was deleted in the diploid strain GVM1. In addition, other candidate genes with SNPs in the promotor or terminator region were investigated for a possible causative role in HMF tolerance. These included glucose/xylose transporter gene *HXT2*, the genes *FAS2*, *GDH3* and *YGL1853* encoding NADH binding domain proteins, and stress response gene *HSP82*, which has been shown to interact with *AST2* and is upregulated upon furfural and acetic acid stress [40]. This revealed that in case of the *AST2*^N406I^ SNP, deletion of the mutant *AST2*^N406I^ allele reduced HMF tolerance, while deletion of the wild type *AST2* allele further enhanced HMF tolerance (**Fig 3A**). For the other SNPs evaluated, no difference in fermentation performance in the presence of HMF between the hemizygous RHA strains containing either the mutant allele or the wild type allele was observed (**S3 Fig**). Sanger sequencing revealed the presence of one *AST2*^N406I^ allele in the GVM0 strain.

The hemizygous RHA strains of GVM1 containing either the mutant or wild type *AST2* allele were also evaluated for tolerance to other inhibitors in comparison with the parent GVM1 strain. The results showed that in YPDX medium *AST2*^N406I^ in comparison with *AST2* significantly improved tolerance to 4.0 g/L furfural (**Fig 3B**). To a smaller extent, this SNP improved tolerance to 4.5 g/L vanillin, although variation in fermentations in the presence of vanillin was quite large (**Fig 3C**). Moreover, in corn cob hydrolysate, unspiked or spiked with 1.0 g/L HMF or 1.0 g/L furfural, the strain with the *AST2*^N406I^ allele also showed a much better fermentation rate compared to the strain with the *AST2* allele (**Fig 3D, 3E and 3F**). The GVM1 strain showed the best fermentation performance in corn cob hydrolysate but not in YPDX medium indicating that in corn cob hydrolysate other factors besides HMF and furfural, that affect the fermentation, have a different dependency on *AST2* activity. Hence, the selection of the transformants after WGT based on fermentation performance in corn cob hydrolysate, likely selected for a transformant with a mutation not only providing superior tolerance to HMF, but also to other fermentation inhibitors present.

### Engineering of *AST2*^N406I^ into *S*. *cerevisiae* strains with different genetic background improves tolerance to multiple inhibitors

**Fig 4:**
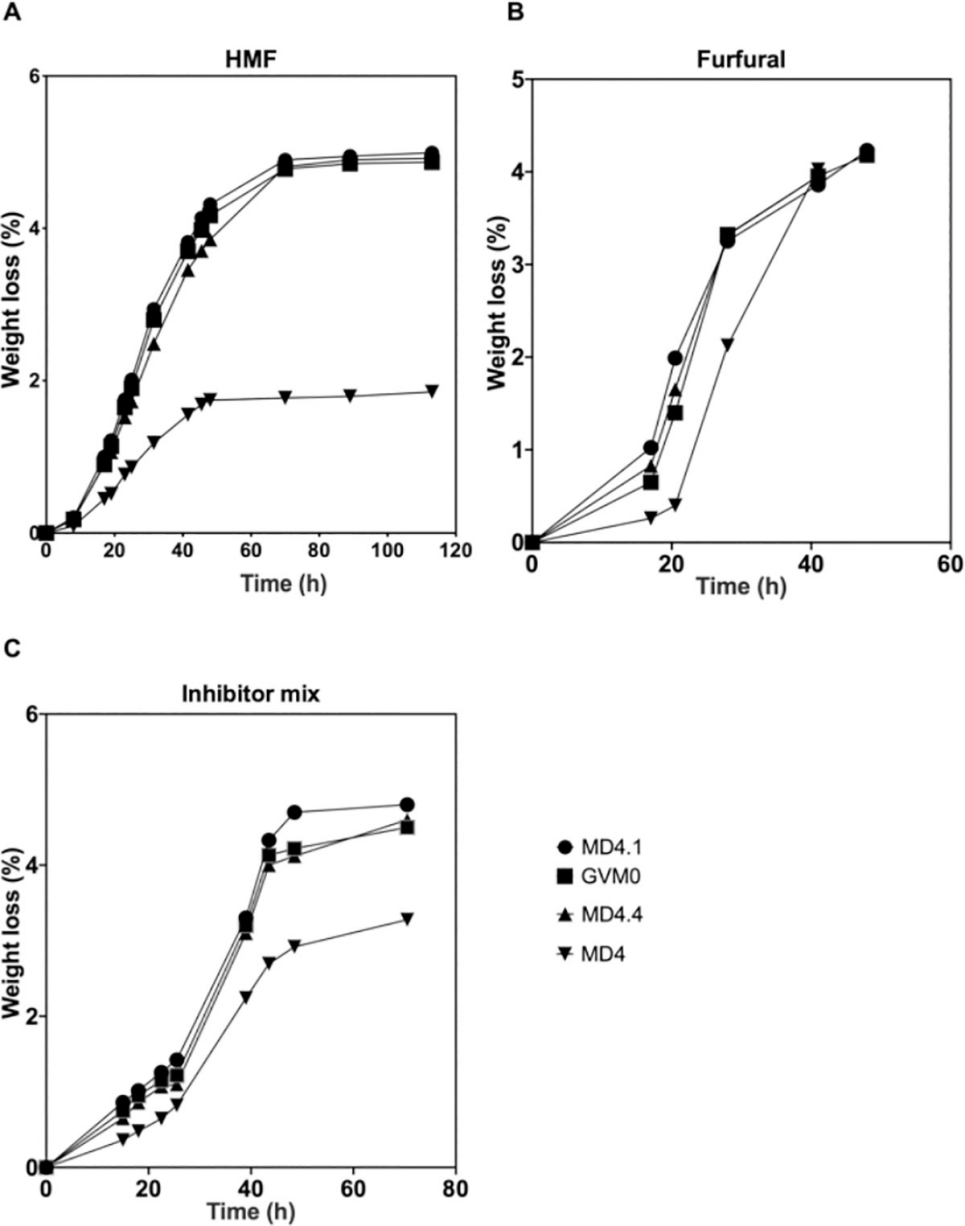
Fermentation performance of MD4, MD4 with one or four copies of *AST2*^N406I^ and the GVM0 WG transformant in the presence of HMF, furfural or a mixture of inhibitors. Small-scale fermentations (10 mL) were performed at 35°C, 350 rpm, initial OD 5.0 in (A) YPDX enriched with 12.0 g/L HMF at pH 5.2, (B) 4.0 g/L furfural at pH 5.2 or (C) a mixture of 2.80 g/L HMF, 1.75 g/L furfural, 0.35 g/L vanillin and 4.20 g/L acetic acid at pH 4.6, to have a starting pH lower than the pKa of acetic acid (4.76). MD4.4: MD4 with four copies of *AST2*^N406I^; MD4.1: MD4 with one copy of *AST2*^N406I^; MD4: with four wild type *AST2* alleles; GVM0: original WG transformant with a single copy of *AST2*^N406I^. Representative result of two biological replicates is shown.

We have engineered in strain MD4, which is tetraploid for *AST2*, one copy (MD4.1) or four copies (MD4.4) of *AST2*^N406I^. The strains obtained were evaluated in YPDX enriched with 12.0 g/L HMF (**Fig 4A**), 4.0 g/L furfural (**Fig 4B**) or a mixture of inhibitors (2.80 g/L HMF, 1.75 g/L furfural, 0.35 g/L vanillin and 4.20 g/L acetic acid) (**Fig 4C**). We also included the original WG transformant of MD4, GVM0, which has also one copy of *AST2*^N406I^ and three *AST2* alleles. The results show that all strains containing at least one *AST2*^N406I^ allele display the same degree of improvement in fermentation performance compared to the MD4 strain. This shows that the *AST2*^N406I^ allele is dominant for conferring tolerance to high concentrations of inhibitors.

To evaluate the general applicability of *AST2*^N406I^ for improving inhibitor tolerance in industrial yeast strains with a different genetic background, we engineered the *AST2*^N406I^ SNP in xylose-utilizing strain TMB3400 and in its original parent strain TMB3000, from which it has been derived by targeted genetic engineering and random mutagenesis [41]. TMB3000 is a highly inhibitor tolerant strain isolated from spent sulfite liquor [42]. It displayed threefold higher NADH-dependent furfural reducing capacity in cell extracts and also a previously unknown NADH-dependent HMF reducing activity [43]. Engineering *AST2*^N406I^ in TMB3400 improved fermentation performance in YPDX with 12.0 g/L HMF, in YPDX with 4.0 g/L furfural, and in YPDX enriched with a mixture of inhibitors (**S4 Fig**). However, introduction of *AST2*^N406I^ in TMB3000, a natural isolate without xylose fermentation capacity, was not beneficial for inhibitor tolerance (**S4 Fig**). Hence, the *AST2*^N406I^ allele may not be effective in all strain backgrounds. Since tolerance to furfural and HMF is most important for fermentation of cellulosic biomass hydrolysates, the *AST2*^N406I^ allele appears most useful for improvement of fermentation performance in engineered xylose-utilizing *S*. *cerevisiae* strains. In all these strains examined the *AST2*^N406I^ allele proved to be effective.

### Isolation of HMF-tolerant transformants using a linear *AST2*^N406I^ fragment

To gain more insight in the mechanism underlying the isolation of transformants with higher inhibitor tolerance using WGT, and especially because of the absence of all nine non-synonymous SNPs in the donor gDNA, we have performed WGT using different types of DNA: gDNA of *C*. *glabrata* (strain JT 26560), gDNA of GVM38 (a transformant with stable, highly improved HMF tolerance, obtained after WGT of MDS130 with *C*. *glabrata* gDNA), and a linear donor fragment with the complete *AST2*^N406I^ allele containing the ORF and 150bp each of its endogenous promoter and terminator, but without any further flanking sequences to lower the chance of insertion by homologous recombination. For this purpose we used a diploid 2G yeast strain, DE-3, originated from the tetraploid MD4 background and with further improved xylose fermentation capacity compared to MD4. Transformation of DE-3 with gDNA from the HMF-tolerant *C*. *glabrata* or GVM38 strains again resulted in a high number of colonies (174 and 90, respectively), whereas transformation of DE-3 with its own gDNA or with water resulted in a low number of colonies (20 and 15, respectively), that never turned out to be stable strains with high inhibitor tolerance after replating on solid nutrient plates with HMF (Table 2). Interestingly, transformation of DE-3 with a linear DNA fragment containing only the *AST2*^N406I^ allele, without any additional sequences for maintenance or integration, resulted in the highest number of HMF tolerant colonies (424). This confirms functional expression of the *AST2*^N406I^ allele in spite of the short linear fragment used. It also appears to indicate that a higher proportion (i.e. 100% for the linear *AST2*^N406I^ fragment) of protective DNA fragments in the donor DNA, compared to the much lower proportion in the gDNA from the tolerant donor strain, leads to a much higher number of positive transformants. Moreover, allele-specific PCR showed that after subculturing in non-selective conditions (absence of HMF), none of the 424 transformants contained the *AST2*^N406I^ mutation. The linear *AST2*^N406I^ fragment must thus have been present in the WG transformants in an unstable form, for instance as eccDNA. Also the 16 stable transformants finally obtained after subculturing in non-selective conditions must have generated an own new mutation conferring higher HMF tolerance, which is in agreement with our previous observation that the *AST2*^N406I^ SNP in the stable HMF-tolerant transformant GVM0 was not derived from the *C*. *glabrata* donor gDNA.

**Table 2:** Number of transformants after WGT of strain DE-3 and selection for improved HMF tolerance. Number of colonies on HMF-containing medium for each transformation condition with or without further subculturing of the colonies is indicated. All YPDX plates contained 6.5% D-glucose and 4.0% D-xylose. All visible colonies on YPDX 7.0 g/L HMF were replated on solid nutrient medium. The transformants that showed growth after replating, were subsequently subcultured in liquid YPDX without HMF for 10 serial transfers and then replated on YPDX with 7.0 g/L HMF.

Source of donor DNA	YPDX 6.0 g/L HMF	YPDX 7.0 g/L HMF		
	Number of colonies	Number of colonies	Number of transformants after replating on YPDX 7.0 g/L HMF	Number of stable transformants after subculturing in YPDX and replating on 8.0 g/L HMF
***C*. *glabrata* gDNA *(*strain JT 26560)**	Full layer of cells	174	40/174	30/40
**gDNA from strain GVM 38**	Full layer of cells	90	15/90	10/15
**Linear DNA fragment with *AST2*** ^**N406I**^	Full layer of cells	424	71/424	16/71
**gDNA from strain DE-3**	Full layer of cells	20	0/20	0/0
**H**_**2**_ **O**	Full layer of cells	15	0/15	0/0

We have selected the 30, 10 and 16 stable transformants of DE-3 obtained after WGT with gDNA from *C*. *glabrata*, gDNA from GVM38, or the linear DNA fragment with *AST2*^*N*406I^, respectively. They were evaluated for HMF tolerance in small-scale fermentations with 12 g/L HMF. This revealed that five of the transformants obtained with the linear DNA fragment containing *AST2*^*N*406I^ (i.e. numbers 50, 51, 52, 53 and 54) showed a better fermentation performance compared to the host strain DE-3 (**S5 Fig**). None of the other WG transformants evaluated showed a better performance, indicating that many transformants isolated on solid nutrient medium with HMF do not show improved fermentation capacity in small-scale fermentations with liquid nutrient medium containing HMF. Possible explanations for the latter are that the 12g/L HMF concentration in the fermentation experiments is higher than the HMF concentrations of 7.0 and 8.0 g/L that were used in the solid nutrient plates for isolation of the transformants, and/or that HMF tolerance on solid nutrient plates and in liquid fermentation medium has somewhat different underlying mechanisms which can make that a WGT strain is more tolerant on plates than in liquid fermentation medium even with the same HMF concentration, and/or that the WG transformants on the solid nutrient plates still contained the protecting element from the donor genomic DNA while it was lost after storage and subculturing of the strains for inoculation in the liquid fermentation medium, and/or that the genomic background of the DE-3 strain contains less elements already supporting HMF tolerance.

### Evaluation of inhibitor tolerance conferred by the corresponding *AST1*^D405I^ mutation

**Fig 5:**
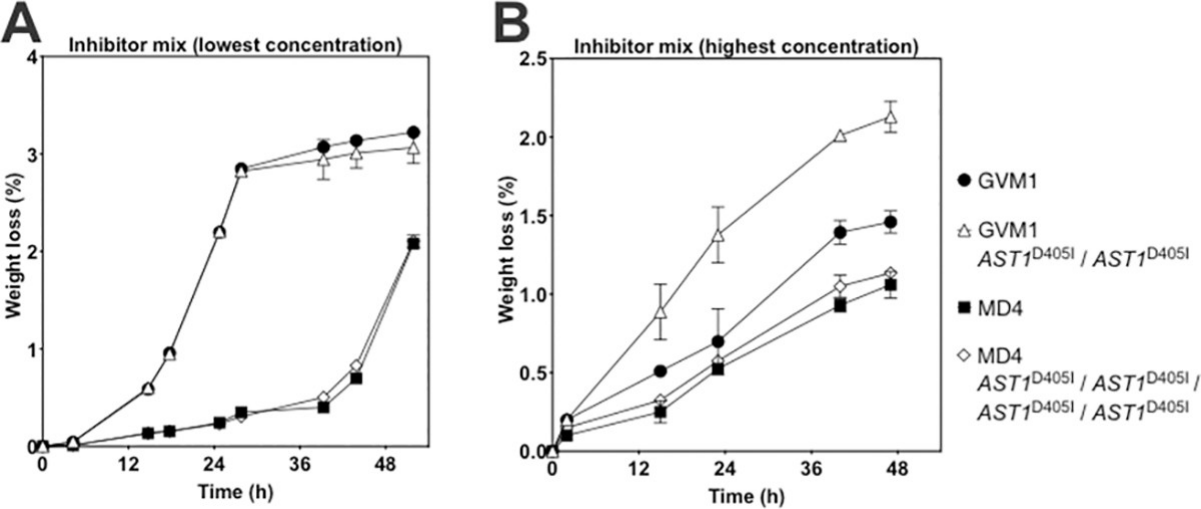
Fermentation performance of GVM1, GVM1 *AST1*^D405I^/*AST1*^D405I^, MD4 and MD4 with 4 copies of *AST1*^D405I^ in the presence of a low- and a high-concentration inhibitor cocktail. Small-scale fermentations (10 mL) were performed at pH 4.6, 35°C, 350 rpm, initial OD_600_ of 5.0 in YPDX with a mixture of (A) 2.80 g/L HMF, 1.75 g/L furfural, 0.35 g/L vanillin and 4.20 g/L acetic acid, or (B) 3.36 g/L HMF, 2.10 g/L furfural, 0.42 g/L vanillin and 5.04 g/L acetic acid. Mean values with standard deviation are shown for four independent transformants of strain GVM1 and MD4, or four technical replicates for strains GVM1 and MD4. Representative result of two biological replicates is shown.

*AST1* is a paralog of *AST2*, also belonging to the quinone oxidoreductase subfamily of the medium-chain dehydrogenase/reductase family. Ast1 and Ast2 have many conserved regions, including the domain downstream from N406 in Ast2 and the corresponding D405 in Ast1. We have engineered into strain GVM1, that contains one copy of *AST2*^N406I^, and MD4, that has only wild type *AST2*, two and four copies of the corresponding mutation *AST1*^D405I^, respectively. The resulting strains were evaluated for inhibitor tolerance in fermentations with YPDX and a mixture of inhibitors (HMF, furfural, vanillin and acetic acid) in low and high concentration. At low inhibitor levels, the *AST1*^D405I^ mutation did not appear to confer any protective effect, neither in the absence of *AST2*^N406I^ in the MD4 strain nor in the presence of *AST2*^N406I^ in the GVM1 strain (**Fig 5A**). On the other hand, in the presence of a high concentration of the inhibitor mix, the GVM1 *AST1*^D405I^/*AST1*^D405I^ strain showed a better fermentation performance compared to the parent GVM1 strain (**Fig 5B**). There was no significant difference in fermentation performance, however, under these conditions for the MD4 and MD4 *AST1*^D405I^/*AST1*^D405I^/*AST1*^D405I^/*AST1*^D405I^ strains (**Fig 5B**). These results indicate that the *AST1*^D405I^ mutation can further enhance the protective effect of *AST2*^N406I^ (which is present in GVM1, but not in MD4) against high concentrations of inhibitors, but that by itself it does not have a detectable effect on inhibitor tolerance under the experimental conditions used.

We also deleted *AST2* and *AST1* in the GVM1 strain, which contains *AST2*^wild type^/*AST2*^N406I^, to further assess their importance for inhibitor tolerance. Deletion of *AST2*^N406I^ in the GVM1 strain reduced tolerance to both low and high inhibitor concentrations (**S6 Fig**), consistent with the previous findings in this paper. Additional deletion of *AST2*^wild type^ did not make a significant difference, consistent with the dominant effect of *AST2*^N406I^. Deletion of a single copy of *AST1* in the GVM1 strain reduced tolerance to both low and high inhibitor levels (**S6 Fig**), further supporting an additional role for *AST1* in inhibitor tolerance. The double *ast1*Δ/*ast1*Δ strain in the GVM1 background could not be obtained.

### Presence of the *AST2*^N406I^ and *AST1*^D405I^ SNPs in whole-genome sequenced *S*. *cerevisiae* strains

We have screened the sequenced genomes of 1011 *S*. *cerevisiae* strains [44] for the possible occurrence of the *AST2*^N406I^ and *AST1*^D405I^ SNPs. While we could not find the *AST1*^D405I^ SNP in any genome, the *AST2*^N406I^ SNP was present in the genome of eight strains, derived from different natural or industrial environments (**S7 Fig**; Materials and Methods). We also evaluated their fermentation capacity in YPDX in the presence of 12 g/L HMF. All these strains showed a similar or better fermentation capacity under these conditions compared to GVM1, except for CLIB564 for which the fermentation capacity was much lower (**S7 Fig**).

### *In vivo* and *in vitro* assessment of aldehyde conversion activity

**Fig 6:**
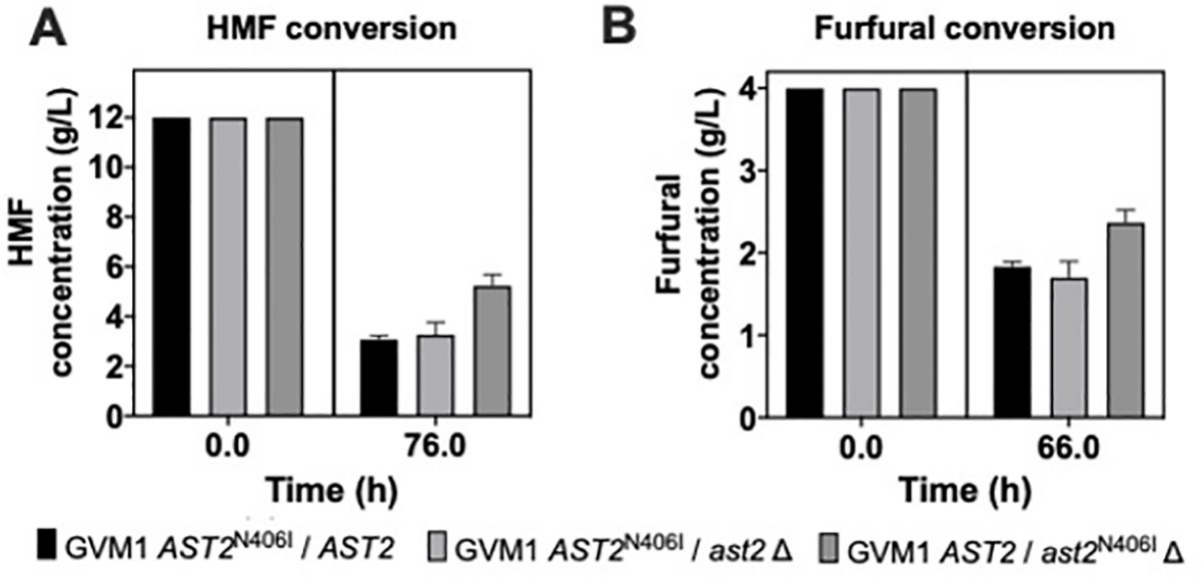
Conversion of HMF and furfural in small-scale fermentations with GVM1 and the hemizygous strains of GVM1 containing either the mutant *AST2*^N406I^ allele or the wild-type *AST2* allele. Small-scale fermentations (10 mL) were performed at pH 4.6, 35°C, 350 rpm, initial OD_600_ of 5.0 in (A) YPDX with 12 g/L HMF or (B) 4 g/L furfural. Subsequent HPLC analysis of fermentation samples obtained (A) in YPDX with 12 g/L HMF at t = 0h and t = 76h, and (B) in YPDX with 4 g/L furfural at t = 0h and t = 66h, revealed that HMF and furfural concentrations had decreased to a greater extent during the course of fermentation by the strains containing the *AST2*^N406I^ allele compared to the strain with the wild-type *AST2* allele. Error bars represent standard deviation.

HPLC analysis revealed that the HMF and furfural concentration in synthetic medium (i.e. YPDX+12 g/L HMF or YPDX + 4 g/L furfural) decreased to a greater extent at the end of fermentation by a strain with one copy of *AST2*^N406I^ (i.e. GVM1 and the hemizygous strain of GVM1 containing only the *AST2*^N406I^ allele) compared to the hemizygous strain of GVM1 harboring only the *AST2* wild-type allele (**Fig 6**). This indicates that *AST2*^N406I^ improves the conversion of both HMF and furfural.

Most genes reported to improve aldehyde tolerance in *S*. *cerevisiae* enhance the in-situ detoxification of these aldehydes by an NAD(P)H-dependent conversion into the corresponding, lesser toxic alcohol. Hence, we have tried to detect aldehyde reductase activity using GST-purified Ast2 and Ast2^N406I^ without and with removal of the GST tag. Furfural and acetaldehyde were used as substrates as well as NADH and NADPH. However, in none of the cases could we detect any significant consumption of NADH or NADPH by the tag-purified proteins.

**Fig 7:**
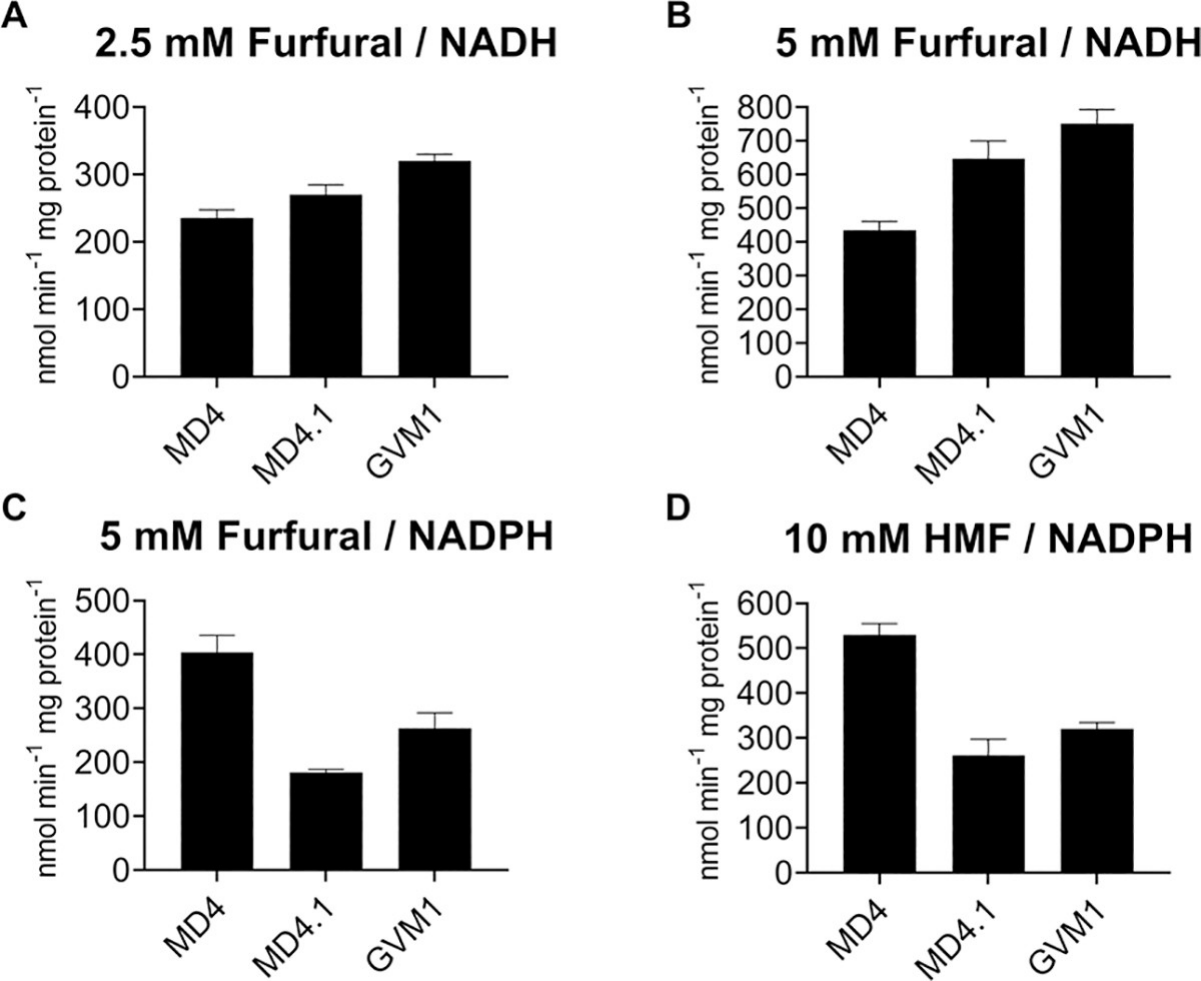
*In vitro* reductase activity with furfural and HMF as substrates in crude cell extracts from the strains MD4, MD4.1 and GVM1, the latter two each containing one copy of *AST2*^N406I^. Reductase activity was measured with NADH or NADPH as cofactor in crude extracts of cells grown on 2% glucose in YP medium until exponential phase. A linear decrease in absorbance was measured at 340 nm for 10 min. For each condition, at least three technical repeats were used. (A) 2.5 mM and 5 mM furfural with NADH, (B) 5 mM furfural with NADPH, (C) 10 mM HMF with NADPH. Error bars represent standard deviation.

We have also measured furfural and HMF reducing activity with NADH and NADPH as cofactors in crude cell extracts. In this case, we were able to detect high reductase activity with furfural and both NADH and NADPH as well as with HMF and NADPH. However, only with the combination furfural (2.5 mM and 5 mM) and NADH could we detect a significant increase in reducing activity in the WGT strains MD4.1 and GVM1 (which both contain the *AST2*^N406I^ allele) compared to the parent strain MD4 (**Fig 7A**). With furfural (5 mM) or HMF (10 mM) and NADPH the reducing activity was actually lower in the WGT strains MD4.1 and GVM1 compared to the parent strain MD4 (**Fig 7B and 7C**).

## Discussion

A major hurdle for economically viable production of bioethanol and bio-based chemicals with lignocellulose hydrolysates is the presence of high levels of inhibitors. In this work we have concentrated on HMF and furfural, generally considered the most toxic inhibitors [45–47]. Total furfural and total furan aldehyde content were also found to be good predictors of yeast fermentation rate in lignocellulose hydrolysates [48,49]. The aldehyde group of these compounds causes damage to DNA, protein and membrane structures, leads to formation of reactive oxygen species (ROS) and inhibits carbon metabolism by inhibition of enzymes [50]. It has been reported that in the presence of both compounds, furfural appears to be converted first into compounds with lower toxicity, before the onset of HMF conversion [48,51].

In this work, we have used two elaborately developed 2G industrial yeast strains, T18 and MD4, with high xylose-utilizing capacity and high inhibitor tolerance. For both strains, we observed strong inhibition of glucose and xylose fermentation in the presence of 1 g/L furfural or 3 g/L HMF. The two strains turned out to be quite tolerant to acetic acid, with only concentrations higher than 7 g/L starting to be strongly inhibitory. Although such concentrations are not common in lignocellulose hydrolysates, the use of cheaper pretreatment methods can lead to concentrations up to 10 g/L acetic acid [49].

Screening of 2526 *S*. *cerevisiae* strains and 17 different non-conventional yeast species for growth on solid nutrient medium with 8 g/L HMF revealed that only 15 strains were able to withstand such a high HMF concentration. Interestingly, nine of these strains were also amongst the 17 most furfural tolerant strains identified. This is most likely due to the similar toxicity that both furan aldehydes exert. Their detoxification mechanisms, including the action of aldehyde reductases, also show considerable overlap between HMF and furfural. The strains originated from a variety of sources although wine yeast strains were overrepresented. It has been reported that during processing of grape must, non-enzymatic browning reactions take place leading to sugar degradation and formation of HMF and furfural under influence of heating [52,53]. The presence of furan aldehydes is a strong indication for high thermal sugar degradation, which may explain the high tolerance of many wine yeast strains.

We performed WGT with gDNA from a *C*. *glabrata* strain, the most HMF tolerant strain in our collection, to select for transformants with higher tolerance to HMF. We found that stable transformants with reliably improved HMF tolerance could only be obtained upon WGT of host strain MD4 with gDNA of an HMF-tolerant donor strain, and not for any control condition used: transformation with water, gDNA of the host strain or of various other non-HMF tolerant strains. This is in agreement with other WGT projects performed in our lab on improvement of acetic acid tolerance and thermotolerance in industrial *S*. *cerevisiae* strains [38,39]. It indicates that the obtained transformants were not contaminants, were not selected because of beneficial mutations already present, or randomly and spontaneously generated, were not strains simply adapted to the presence of HMF or mutations induced by the donor DNA acting as a random mutagen. We initially expected to find evidence for homologous recombination between the gDNA of the host and donor strain with *C*. *glabrata* DNA fragments inserted in the host genome. However, in spite of meticulous scrutiny, we could not find any, not even very small fragments of the *C*. *glabrata* gDNA inserted in the genome of the host strain MD4 in the HMF tolerant transformants. Instead, only very few non-synonymous SNPs could be detected when the genome sequence of the WG transformant and its host strain were rigorously compared. Even more surprisingly, none of these SNPs apparently originated from the donor gDNA, by e.g. recombination between homologous sequences, since also none of the SNPs was present in the *C*. *glabrata* genomic DNA. This was also true for the causative *AST2*^N406I^ mutation that we subsequently identified among the non-synonymous SNPs. This surprising finding was also made in two other research projects carried out at the same time in our research group on the isolation of WG transformants with either higher acetic acid tolerance or higher thermotolerance [38,39].

We have performed multiple control experiments, which all indicate that the introduction by WGT of gDNA from a strain with a superior trait of interest, e.g. higher tolerance to HMF, is essential to obtain stable WG transformants also displaying (to a certain extent) the superior trait. Hence, the only plausible explanation that we can envisage is that part of the foreign gDNA in some way transiently protects the host strain against the stress condition. Foreign DNA fragments with a protective gene could be maintained as extrachromosomal DNA (eccDNA) for a number of generations under the selective conditions. Ample evidence is available that yeast cells can easily generate eccDNA and also that it can be actively expressed [54,55]. The protective eccDNA would allow the transformants to multiply for a few generations, creating more time and opportunity to generate spontaneous mutations in the host strain that confer higher tolerance to the stress condition. This explains the very low number of SNPs detected and the presence of just a single causative SNP, that is absent from the donor gDNA. Likely, many more transformants received a fragment of the donor gDNA, but when it did not confer higher tolerance to the stress condition, it did not provide any advantage to the host strain and such transformed cells therefore could not survive. On the other hand, cells that received a DNA fragment with an element conferring protection to the stress condition, could start to multiply and keep multiplying as long as they maintain the protective DNA fragment, and thus had a much higher chance of generating a spontaneous rescuing mutation. Possibly, many more transformants have generated spontaneous mutations, but without generation of a mutation conferring higher tolerance to the stress condition, these transformants could not survive, at least not permanently in a stable form. Once the host strain has generated a spontaneous rescuing mutation, the heterologous gDNA fragment is no longer needed and can easily get lost. Future work should allow to assess whether this hypothesis on the underlying mechanistic explanation of our observations is correct.

When we transformed the DE-3 strain with a linear DNA fragment containing the superior *AST2*^N406I^ allele, we obtained the highest number of positive transformants upon selection for high HMF tolerance. This is likely due to the fact that all DNA fragments entering the host cells during WGT carry a protective element. When transformation, on the other hand, is performed with gDNA from an HMF tolerant strain, only a minority of the DNA fragments entering the host strain will confer a protective effect, explaining the lower number of transformants. We previously observed that the strain GVM0, obtained after WGT with the heterologous *C*. *glabrata* gDNA, did not contain any gDNA fragments of the donor strain. Similarly, after transformation with the linear *AST2*^N406I^ fragment, none of the transformants contained the *AST2*^N406I^ SNP. Hence, as in the case of WGT with the *C*. glabrata gDNA, the transformants obtained after transformation with the linear *AST2*^N406I^ fragment, apparently also generated another spontaneous protective mutation in their own gDNA.

We have identified the *AST2*^N406I^ SNP as the only causative mutation. Relatively little is known about the function of the *AST2* gene product. Based on sequence homology, it has been classified as a member of the quinone oxidoreductase subgroup in the superfamily of medium-chain dehydrogenase/reductases (MDR) [56]. *AST2* has a close paralog, *AST1*, that arose from the whole genome duplication of *S*. *cerevisiae*. Ast1 is a peripheral membrane-associated protein that is involved in trafficking of the plasma membrane H^+^-ATPase to the plasma membrane and its association to lipid rafts [57,58]. No studies have been reported on Ast2 and it is unclear whether it performs a similar function as Ast1 in control of H^+^-ATPase trafficking. Based on sequence homology, *AST2* and *AST1* encode putative aldehyde reductase/alcohol oxidases [56]. Such an enzymatic activity would also fit with previous reports that genes encoding aldehyde reductases improve furfural and HMF tolerance in an NAD(P)H-dependent manner [12–15,17–19,59,60]. The underlying explanation for the beneficial effect of at least some aldehyde oxidoreductases has been that they convert HMF and furfural into the corresponding alcohols, which have much lower toxicity [6]. *S*. *cerevisiae* and *P*. *pastoris* strains obtained by adaptive laboratory evolution for higher tolerance to furfural also showed higher conversion capacity for these compounds [47,61,62]. Tolerance to furfural can also be increased by overexpression of *ADH7*, *YKL071W* and *ARI1*, which encode reductases involved in furfural reduction [59,60]. High furaldehyde reductase activity was also found in a highly inhibitor-tolerant *S*. *cerevisiae* natural isolate from spent sulfite liquor [63]. Also improved vanillin tolerance in *S*. *cerevisiae* mutants has been correlated with enhanced vanillin reducing activity [64]. *E*. *coli* and *Zymomonas mobilis* strains with enhanced furan aldehyde reductase activity were also shown to display higher HMF and furfural tolerance and improved fermentation rates in lignocellulose hydrolysates [65]. Not all oxidoreductase genes that are significantly induced in the presence of furfural support higher HMF reducing activity in cell extracts of strains in which these genes are individually overexpressed. Among these genes, *ADH6* and *ADH7* were the most effective in supporting higher NADPH-dependent activity and *SFA1* for higher NADH-dependent activity [12]. A similar improvement of HMF and furfural tolerance was reported in xylose utilizing strains overexpressing *Pichia stipitis* xylose reductase [66]. On the other hand, some NADPH-dependent furfural reductases with low Km for NADPH actually reduced furfural tolerance and many furfural-induced oxidoreductase-encoding genes were inefficient in enhancing furfural tolerance upon overexpression [67]. The yeast oxidoreductases likely have widely different substrate specificities. This is illustrated by the fact that overexpression of *ADH6* not only improves furfural and HMF tolerance but also vanillin tolerance [22], while in our work the *AST2*^N406I^ SNP improved both furfural and HMF tolerance but appeared to have only little effect on vanillin tolerance.

Combination of the *AST2*^N406I^ mutation with the corresponding mutation *AST1*^D405I^ further improved inhibitor tolerance under certain conditions, while deletion of *AST1* reduced inhibitor tolerance. These results might support the conclusion that both *AST1* and *AST2* encode functional oxidoreductases. This would also fit with the dominant character of the *AST2*^N406I^ allele. However, no experimental evidence for such an enzymatic function has been reported for Ast2 or Ast1 up to now and we were also unable to demonstrate aldehyde reductase activity *in vitro* with tag-purified Ast2 and Ast1 proteins. On the other hand, we could detect *in vitro* reductase activity with crude cell extracts using furfural and NADH as well as HMF, NADH or NADPH as substrates. Only with furfural and NADH did we observe a significant increase in reducing activity in the WGT strains MD4.1 and GVM1 (which contain the *AST2*^N406I^ allele) compared to the parent strain MD4. This could be consistent with the *AST2*^N406I^ gene product displaying higher intrinsic catalytic activity. However, with the combinations HMF, NADH or NADPH we observed a decrease in reducing activity in the WGT strains MD4.1 and GVM1 compared to the parent strain MD4. This is not consistent with higher catalytic activity of the *AST2*^N406I^ gene product and does not fit with the higher HMF tolerance and faster HMF reducing rate *in vivo*. The discrepancy between the activity measurement in crude cell extracts and with tag-purified Ast2 proteins might be due to a requirement for one or more additional small molecule or protein cofactors to support catalytic activity of Ast2. On the other hand, it could also indicate that Ast2 does not have inherent aldehyde reductase activity but acts in a different way, for instance as a regulator or subunit of genuine aldehyde reductases. This may explain the inconsistent results that we obtained for reductase activity in crude cell extracts with HMF as substrate. Future research will have to elucidate the precise action mechanism of Ast2 and the mutant Ast2^N406I^ protein. Overexpression of aldehyde reductases may further improve tolerance to furaldehyde inhibitors in the 2G yeast strains, harboring the *AST2*^N406I^ and *AST1*^D405I^ alleles. On the other hand, the generation of sufficient redox power may become a limiting factor for further enhancement of tolerance to furan aldehydes through their conversion to the corresponding alcohols.

TMB3000 is a highly inhibitor tolerant strain isolated from spent sulfite liquor [42]. It displayed threefold higher NADH-dependent furfural reducing capacity in cell extracts and also a previously unknown NADH-dependent HMF reducing activity [43]. Compared to a non-inhibitor tolerant strain, the higher NADH-coupled conversion of HMF into the corresponding alcohol was shown to be solely responsible for its higher ethanol productivity. The NADH-coupled HMF reduction of this strain is interesting since reduction of HMF was previously always described as being NADPH-coupled, unlike reduction of furfural into furfuryl alcohol that has always been described as being NADH-coupled [12,43,68–70]. Since the *AST2*^N406I^ allele did not improve inhibitor tolerance in the TMB3000 strain, the unusual NADH-coupled HMF reduction in TMB3000 may be due to a mutation in *AST2* or in another oxidoreductase-encoding gene that makes the contribution of *AST2*^N406I^ superfluous, as was observed for the additional alleles of *AST2*^N406I^ introduced in the MD4 strain containing already a single *AST2*^N406I^ allele.

It is also not clear how the *AST2*^N406I^ allele improves acetic acid tolerance. One possible explanation might be that high acetic acid levels lead to higher acetaldehyde levels, which are then converted by Ast2 into ethanol, although we could not detect such activity with the purified Ast2 and Ast2^N406I^ proteins. Acetaldehyde is much more toxic to yeast than ethanol [71]. In lignocellulose hydrolysates a complex and variable range of inhibitory compounds is present. The observation that the GVM1 strain showed the best fermentation performance in corn cob hydrolysate but not in YPDX medium indicates that in corn cob hydrolysate other inhibitors besides HMF, furfural and vanillin, have a different dependency on *AST2* activity. Hence, the *AST2*^N406I^ allele might even be more beneficial in real biomass hydrolysates compared to synthetic media. Since cheaper pretreatment methods result in higher levels of inhibitors in lignocellulose hydrolysates, availability of 2G bioethanol yeast strains with higher inhibitor tolerance will allow the use of cheaper pretreatment methods and thus enhance the economic viability of 2G bioethanol production. Furthermore, higher concentrations of inhibitors will make it more difficult for other yeast species or bacteria to thrive in a 2G bioethanol fermentor, reducing the risk of yield losses due to contaminating micro-organisms.

The presence of the *AST2*^N406I^ SNP in the genome of eight *S*. *cerevisiae* strains isolated from diverse natural sources appears to provide further indirect support for the beneficial effect of this mutation for survival of *S*. *cerevisiae* in specific niche environments. It makes the *AST2*^N406I^ allele a naturally occurring allele, which may be beneficial for the development of naturally cisgenic industrial *S*. *cerevisiae* strains for diverse applications.

## Conclusions

In this work we have demonstrated the efficacy of WGT for improvement of selectable phenotypes in industrial yeast strains. Surprisingly, very few genetic modifications were present in the superior transformants and all SNPs detected were unrelated to the genome sequence of the donor strain. This raises intriguing questions about the mechanistic explanation for the requirement of gDNA from a superior host strain to successfully obtain stable WG transformants by WGT. Our findings make WGT a very useful technology for industrial strain improvement because it minimizes the risk of side-effects on other traits. The superior *AST2*^N406I^ allele identified confers tolerance to the toxic aldehydes HMF and furfural, and to acetic acid and other inhibitors in lignocellulose hydrolysates when introduced in various xylose-utilizing *S*. *cerevisiae* strains, apparently making it an excellent tool for improvement of inhibitor tolerance in cellulosic yeast strains.

## Materials and methods

### Yeast strains and cultivation media

Yeast strains used in this work are listed in **S1 Table**.

### Small-scale fermentations in corn cob hydrolysate and in synthetic medium

Inhibitor tolerance of T18 and MD4 was evaluated in corn cob hydrolysate (see **S2 Table** for composition), that was spiked with a range of industrially-relevant inhibitor concentrations. After preculture of the yeast strains for 48 h at 30°C with shaking at 200 rpm in YPD2% (10 g/L yeast extract, 20 g/L bacteriological peptone, 2% D-glucose) up to stationary phase, small-scale (10 mL) semi-anaerobic fermentations with MD4 and T18 were performed at pH 5.2, 35°C, shaking at 350 rpm, and a yeast inoculum OD 5.0. Weight loss of the fermentation tubes, which is correlated with CO_2_ production during conversion of glucose and xylose into ethanol, was measured continuously, or sampling at different timepoints was performed to analyse sugar and inhibitor concentrations by HPLC.

All other fermentations were also performed at pH 5.2, 35°C, shaking at 350 rpm, and a yeast inoculum OD 5.0, either in 1) YPD6.5% with 8 g/L HMF for screening of fermentation capacity of the most HMF tolerant strains from our yeast strain collection, in 2) YPD6.5% X4.0% (4% D-xylose) with 6 g/L or 12 g/L HMF, or corn cob hydrolysate (see **S2 Table** for composition) enriched with 0.0 g/L, 0.6 g/L, 1.0 g/L or 3.0 g/L HMF to evaluate HMF tolerance in fermentations of the WG transformants of MD4 and the segregants of GVM0, in 3) YPD6.5% X4.0% with 12 g/L HMF to evaluate the genetic modification in GVM1 causative for enhanced HMF tolerance, and in 4) YPD6.5% X4.0% with 12 g/L HMF, or corn cob hydrolysate 2 enriched with 0.0 g/L, 0.6 g/L, 1.0 g/L or 3.0 g/L HMF to evaluate the effect of *AST2*^N406I^ in MD4 for tolerance of yeast fermentation capacity to different inhibitors and stress factors.

### Screening of yeast strain collection

A yeast strain collection of 2526 *S*. *cerevisiae* strains and 17 non-conventional yeast species previously reported as displaying high tolerance to HMF during growth on solid nutrient medium [72], was screened for their level of HMF tolerance by evaluating growth after 48 h at 30°C on solid synthetic nutrient medium (YPD2%) with 8 g/L HMF. The non-conventional yeast species screened were *Candida glabrata*, *Metschnikowia reukaufii*, *Kluyveromyces marxianus* (2 strains), *Brettanomyces bruxellensis*, *Pachysolen tannophilus*, *Ambrosiozyma monospora*, *Scheffersomyces stipitis*, *Saccharomyces servazii* (3 strains), *Zygosaccharomyces bailii* (4 strains*)*, *Torulaspora delbrueckii*, *Issatchenkia orientalis*, *S*. *kudriazevii* (2 strains), *Pichia kluyverii*, *Debaryomyces hansenii*, *Meyerozyma guilliermondii*, *Pichia membranifaciens* and *Pichia anomala*.

### Whole-genome transformation and selection of transformants

MD4 was whole-genome transformed with gDNA from *C*. *glabrata* strain JT26560, and *S*. *cerevisiae* strains JT25869, JT23146, JT21620, JT23341, MD4, S288C, JT25416, JT25880, JT22277 and JT22689. For isolation of gDNA, yeast cells were suspended in 200 μl water and mixed with glass beads (0.45 mm) in 2 ml screw cap tubes into which 200 μl PCI solution [45.5% (v/v) phenol pH 4.2, 43.6% (v/v) chloroform, 1.8% (v/v) isoamyl alcohol, 9.1% (v/v) sodium dodecyl sulfate] was added. Cells were lysed with a FastPrep-24® Classic Instrument for 20 s at 6.0 M/s, and cell lysate was centrifuged (10 min at 14,000 rpm). 200 μl clear supernatant was mixed with 1000 μl ice-cold 99.8% ethanol, vortexed and stored at -20°C for 1 h. The pellet was washed with 70% ethanol, resuspended in 50 μl water and sheared with the FastPrep for 60 s at 6.5 M/s to increase the fraction of smaller gDNA fragments. More than 50% of the gDNA fragments were between 100 and 10,000 bp. For WGT, 5 μg gDNA was transformed into tetraploid strain MD4 via electroporation. After 4 h recovery in 1:1 YPD2% and 1M D-sorbitol, transformants were plated on YPD6.0% X4.5% with 2.5 g/L HMF and incubated at 30°C for 72 h. Transformants obtained were restreaked on YPD6.0% X4.5% plates with 2.5 g/L or 4.0 g/L HMF.

### Transformation of *S*. *cerevisiae*

Yeast strains were transformed for introduction of plasmids for CRISPR/Cas9 targeting, to perform RHA or for whole-genome transformation. This was either achieved by electroporation according to Benatuil et al. [73] or by transformation according to Gietz and Schiestl [74].

### Sporulation of strain GVM0

The tetraploid strain GVM0, obtained by WGT of MD4 with gDNA of *C*. *glabrata*, was sporulated to obtain diploid segregants. For that purpose, the strain was first cultured overnight in YPD2% at 30°C and 200 rpm, subsequently inoculated into 30 ml YPD2% at OD 1 and cultivated for 6h at 30°C and 200 rpm until exponential phase. Cells were washed with water and plated on two solid sporulation media (1% potassium acetate, 0.25% yeast extract, 0.1% D-glucose at pH 6) and CSH (1% potassium acetate, 0.05% dextrose, 0.10% yeast extract). After lyticase treatment for 3 min at RT, single spores were isolated with a dissection microscope (Singer instruments).

### Genomic DNA isolation, whole-genome sequencing and bio-informatics analysis

gDNA of strains MD4 and GVM1 was isolated with the MasterPure^TM^ Yeast DNA Purification Kit (Lucigen) and submitted to whole-genome sequence analysis (Illumina) with 125 bp paired-end reads. DNA sequences were mapped by using the NGSEP pipeline (version 3.3.1) [75]. Bowtie 2 [76] was used to map the genome of MD4 and GVM1 against that of S288C (version R64-2-1 at SGD). Parameters for variant calling were [-runRP -runRep -runRD -maxBaseQS 30 -minQuality 40 -maxAlnsPerStartPos 2 -knownSTRs <STR_file>]. Tandem Repeats Finder [77] was used to generate an STR file of each reference genome. The combined.vcf file was filtered using parameter [-q 40] and functional annotation of genomic variants was performed with NGSEP. Further filtering was achieved with in-house scripts. In this way, a list of genomic variations between MD4 and GVM1 was generated, which consisted of nine heterozygous non-synonymous SNPs.

### Screening of sequenced *S*. *cerevisiae* whole genomes for presence of *AST2*^N406I^

The *AST2*^N406I^ allele was present in the genome of a wine yeast (CBS5835), a natural isolate from oak (EXF7145), a natural isolate from wax on rock surface (NCYC3985), an isolate from grape must (Lib 73), two isolates from dairy cheese camembert (CLIB564 and CLIB558), an isolate from Japanese kefyr grains (CBS2421) and a soil isolate from Taiwan (EN14S01).

### Reciprocal hemizygosity analysis (RHA)

RHA was performed with strain GVM1. For this purpose, a nourseothricin (clonNAT) cassette was amplified with Q5 polymerase in a medium containing 4 μl Q5 buffer, 4 μl GC enhancer, 1.6 μl dNTPs (10 mM), 1 μl forward primer (10 μM), 1 μl reverse primer (10 μM), 0.2 μl Q5® HF polymerase (New England BioLabs, NEB) and 1 ng p77 plasmid (in a 50 μl reaction volume) from plasmid pTOPO-A1-G2-B-NAT-P-G2-A2(p77) with specific primer tails for the 9 non-synonymous SNPs identified in GVM1 after WGT of MD4. PCR amplification was performed as follows: 4 min at 98°C, followed by 30 cycles consisting of 30 s at 98°C, 30 s at 70°C and 1 min at 72°C, followed by 5 min at 72°C. The cassette generated was transformed into GVM1 by the Gietz protocol to delete each time one allele of the heterozygous gene containing a non-synonymous SNP. Transformants were subsequently plated on YPD2% with 100 μg/ml nourseothricin, and evaluated for deletion of either the wild type or the mutant allele via allele-specific PCR with TaqE polymerase [2 μl Buffer E, 2 μl dNTPs (10 mM), 1 μl forward primer (10 μM), 1 μl reverse primer (10 μM), 0.5 μl TaqE polymerase, 1μl gDNA (100 ng/μl) in 20 μl total volume]. PCR amplification was carried out as follows: 4 min at 94°C, followed by 30 cycles of 25 s at 94°C, 25 s at 55°C, and 45 s at 72°C), followed by 5 min at 72°C. Correct deletion of the two alleles was confirmed by Sanger sequencing (Mix2Seq at Eurofins). The list of primers used in this work is shown in **S3 Table**.

### CRISPR/Cas9 genome editing

CRISPR/Cas9 genome editing was performed to introduce multiple copies of *AST2*^N406I^ in strains MD4, GVM1, TMB3400 and TMB3000; and also to introduce *AST1*^D405I^ in MD4. To perform CRISPR/Cas9 in *S*. *cerevisiae* strains, guide RNAs (gRNAs) were designed based on the whole-genome sequence data of the strains to be modified. The CRISPR/Cas9 plasmids (from *Streptococcus pyogenes*) used were modified from [78] as follows. The hCas9 plasmid (Addgene #41815) was modified with a KanMX cassette in order to select transformants on solid nutrient plates with geneticin (plasmid p51-KanMX). The gRNA_Cloning Vector (Addgene #41824) was modified with a NatMX cassette in order to select transformants on solid nutrient plates with nourseothricin (plasmid p59-NAT). Based on on-target activity, aspecific cleaving (determined via a blast search of 12bp from the 3’ end of the gRNA followed by NGG, NGA or NAG), proximity to *AST2*^N406I^ or *AST1*^D405I^, absence of a stretch of five or more thymines, we selected the most efficient gRNA, 5’–TTATTCCTGGAAAAATTTCA– 3’, to target *AST2* and 5’—TATAAGAAAATGCTTCTTTA—3’ to target *AST1*. A linear donor fragment containing the *AST2*^N406I^ or *AST1*^D405I^ mutation was used to repair the double strand break after CRISPR/Cas9 targeting.

After restriction digestion with XhoI (NEB) and EcoRV (NEB), the gRNA was cloned in plasmid p59 using Gibson assembly (NEB), in a reaction with 50 ng plasmid and 3 times molar excess of the gRNA insert, followed by incubation at 50°C for 1 h. Two μl of the ligation mixture was transformed into DH5alpha *Escherichia coli* cells that were previously made competent with RbCl treatment [79]. Cells were incubated for 30 min on ice, heat shocked for 45 s at 42°C, and incubated again for 5 min on ice. Next, 1 ml LB medium (10 g/L tryptone (Oxoid), 5 g/L yeast granulated extract (Merck), and 1 g/L NaCl 99.5% were added, and the cells were incubated at 37°C and 300 rpm for 1 h. Subsequently, the transformed *E*. *coli* cells were plated on solid LB plates with 100 μg/ml ampicillin, and incubated overnight at 37°C. Next, plasmid p59-NAT-gRNA-AST2 was purified with NucleoSpin® Plasmid EasyPure (Macherey-Nagel). Thereafter, p51KanMX and subsequently p59-NAT-gRNA-AST2 or p59-NAT-gRNA-AST1, as well as the linear *AST2*^N406I^ or *AST1*^D405I^, were transformed into strains MD4 and GVM1 via electroporation. After loss of the two plasmids by subculturing under non-selective conditions, the transformants were analyzed by allele-specific PCR and Sanger sequencing.

### HPLC

For HPLC, a Bio-Rad Aminex HPX 87H 300X7 8mm column was used. The eluant was H_2_SO_4_.

### Enzyme activity

*AST* genes were N-terminally fused to GST in the pGEX4T-1 plasmid (GE Healthcare). A Factor Xa protease sequence was included for clean-cutting purified Ast protein from the GST moiety. Subsequent attempts to measure aldehyde reductase activity with purified Ast2^wt^ and Ast2^N406I^ made use of two approaches. They both included recombinant protein expression in BL21 *E*. *coli* cells using 0.5 mM IPTG induction overnight at 20°C in LB medium. Cell lysate was obtained by incubating induced *E*. *coli* cells in digestion buffer (50 mM Tris-HCl pH 7.5, 150 mM NaCl, 1 mM EDTA pH 8, 5% glycerol, 1% Triton X-100, 1 mM EDTA and 5 mg/mL lysozyme) followed by three sonication events of 10 s each with an intermediate pause on ice. In the first approach, enzyme activity was then measured directly with Ast proteins bound to Glutathione Sepharose 4B. For that purpose, cell lysates containing Ast recombinant protein were added to GSH-coated beads (GE Healthcare), which were rotor incubated for at least 2 h in chilled conditions and washed three times with washing buffer (50 mM Tris-HCl pH 7.5, 150 mM NaCl, 1 mM EDTA pH 8, 5% glycerol). Correct fusion protein expression was always verified by SDS-PAGE followed by Coomassie Blue Brilliant staining. Ast-bound beads were incubated in reaction buffer (50 mM Tris-HCl pH 7.5, 150 mM NaCl, 1 mM ZnCl_2_) at 30°C. The reaction was initiated by adding either 10 mM furfural or 10 mM acetaldehyde as substrate and 0.8 mg/mL NADH or NAPDH as cofactor. Every 5 min, resuspended beads were spun down and the supernatant sampled for determination of OD at 340 nm. However, no consumption of NADH or NADPH could be detected. In the second approach, the Ast proteins were cut from GST to rule out compromised enzyme activity by steric hindrance. Recombinant Ast protein from cell lysate obtained with 1.5 L induced *E*. *coli* suspension was column purified (GSTrap^TM^ Fast Flow, 1 mL, GE Healthcare) and overnight digested with 25 μg/mL Factor Xa. Purification and digestion protocols were used as recommended by GE Healthcare. The presence of full-length Ast protein in eluate fractions was confirmed by SDS-PAGE. However, no enzymatic activity could be detected, even when high protein concentrations (> 200 μg/mL) were added to the reaction mixture.

In order to assess aldehyde reductase activity in crude cell extracts, the cells were grown on 2% glucose in YP medium until exponential phase. Cells were harvested and washed twice with ice-cold 25 mM MES-buffer, pH 6. Next, cells were mechanically lysed in lysis buffer (50 mM Na_2_HPO_4_/NaH_2_PO_4_ (pH 7), 5% glycerol, 1% Triton X-100) supplemented with protease inhibitor cocktail (Roche) plus 1 mM PMSF to prevent proteolytic degradation. From the cleared supernatant, protein concentrations were measured using Pierce™ reagent (ThermoFisher). To measure activity, assay buffers were prepared containing 100 mM Na_2_HPO_4_/NaH_2_PO_4_, pH 7, supplemented with either 1 mM NADH or 1 mM NADPH to which 0.1 mg protein/mL cell extract was added. From this, 200 μL/well of assay buffer was transferred to a 96-well plate which was first allowed to temperature equilibrate for 10 min at 30°C. For each condition, at least three technical repeats were used. When a steady-state absorbance at 340 nm was measured, either 2.5 or 5 mM furfural, or 10 mM HMF was added to the reaction mixture to start the reaction. Linear decrease in absorbance was measured over the span of 10 min from which the reaction rate was calculated.

## Supporting information

S1 Fig Fermentation performance of WG transformants of MD4 in HMF-enriched corn cob hydrolysate. Evaluation of the fermentation capacity of WG transformants in the presence of HMF in small-scale fermentations (10 mL) in corn cob hydrolysate enriched with (A) 0.0 g/L or (B) 1.0 g/L HMF, pH 5.2, 35°C, 350 rpm and initial OD 5.0. The GVM0 strain and Transformants 2 to 9 were obtained by transformation of strain MD4 with gDNA of *C*. *glabrata* strain JT26560, Transformant 10 with gDNA from wine yeast DBVPG 1552 (JT25869), Transformant 11 with own gDNA of MD4, Transformant 12 with water, and Transformant 13 with gDNA from lab strain S288c. The three latter conditions never resulted in strains with stable improved HMF tolerance, neither when grown on nutrient plates nor when evaluated in small-scale fermentations. Three biological replicates were performed for the strains MD4, JT26560 and GVM0. Two technical replicates were performed for strain MD4. All other strains were evaluated once.
(TIFF)

S2 Fig Fermentation performance of tetraploid WG transformant GVM0 and its diploid segregant GVM1 in HMF-enriched corn cob hydrolysate. Small-scale fermentations (10 mL) were performed in corn cob hydrolysate enriched with 0.0 g/L or 1.0 g/L HMF, pH 5.2, 35°C, 350 rpm and initial OD 5.0. Representative result of two biological replicates is shown.
(TIFF)

S3 Fig Fermentation performance of the two hemizygous strains of WG transformant GVM1, containing either the mutant or wild-type allele, for eight genes in which a non-synonymous SNP was present, for *HXT2*, *FAS2*, *GDH3*, *YGL185C*, which all contained insertions in their promoter, and for *HSP82*, which contained a synonymous mutation in its ORF. Small-scale fermentations (10 mL) were performed in YPDX medium enriched with 12.0 g/L HMF, pH 5.2, 35°C, 350 rpm and initial OD 5.0. Mean values with standard deviation are shown for four independent transformants of strain GVM1, or four technical replicates for strains GVM1. The experiment was performed once.
(TIFF)

S4 Fig Fermentation performance of the industrial yeast strains TMB 3000 and TMB3400 engineered to contain a single copy of *AST2*^N406I^. Small-scale fermentations (10 mL) were performed at 35°C, 350 rpm, initial OD 5.0 in YPDX, enriched with 12.0 g/L HMF at pH 5.2 (A, B), 4.0 g/L furfural at pH 5.2 (C, D) and an inhibitor mixture of 2.80 g/L HMF, 1.75 g/L furfural, 0.35 g/L vanillin and 4.20 g/L acetic acid at pH 4.6 (E, F). Mean values with standard deviation are shown for three independent transformants of strains TMB 3000 and TMB 3400, or two technical replicates for strains TMB 3000 and TMB 3400. The experiment was performed once.
(TIFF)

S5 Fig Fermentation performance of WG transformants of DE-3, obtained with linear DNA fragment containing *AST2*^N406I^, in YPDX in the presence of HMF. Evaluation of the fermentation performance of WG transformants of DE-3 (i.e. Transformant 50, 51, 52, 53, 54), selected for improved HMF tolerance, in small-scale fermentations (10 mL, pH 5.2, 35°C, initial OD 5.0, 350 rpm in synthetic YPDX medium enriched with 12 g/L HMF). The strains were evaluated once except for strain DE-3 for which two technical replicates were used. The experiment was performed once.
(TIFF)

S6 Fig Fermentation performance of GVM1, GVM1 *AST2*^*wild-type*^*/ast2*^N406I^Δ, GVM1 *ast2*ΔΔ and GVM1 *ast1*Δ for inhibitor tolerance. Small-scale fermentations (10 mL) were performed at pH 4.6, 35°C, 350 rpm, initial OD_600_ of 5.0 in YPDX with a mixture of (A) 2.80 g/L HMF, 1.75 g/L furfural, 0.35 g/L vanillin and 4.20 g/L acetic acid, or (B) 3.36 g/L HMF, 2.10 g/L furfural, 0.42 g/L vanillin and 5.04 g/L acetic acid. Mean values with standard deviation are shown for three independent transformants of the derivatives of GVM1, or three technical replicates for the strain GVM1. The experiment was performed once.
(TIFF)

S7 Fig Fermentation performance of GVM1 and eight *S*. *cerevisiae* strains that also contain the *AST2*^N406I^ mutation. Small-scale fermentations (10 mL) were performed in YPDX medium enriched with 12.0 g/L HMF, pH 5.2, 35°C, 350 rpm and initial OD 5.0. Strains depicted are GVM1, a wine yeast (CBS5835​), a natural isolate from oak (EXF7145), a natural isolate from wax on rock surface (NCYC3985), an isolate from grape must (Lib 73), two isolates from dairy cheese camembert (CLIB564 and CLIB558), an isolate from Japanese kefyr grains (CBS2421) and a soil isolate from Taiwan (EN14S01). The strains were evaluated once except for strain GVM1 for which two technical replicates were used. The experiment was performed once.
(TIFF)

S1 Table List of yeast strains used in this study. (DOCX)

S2 Table Sugar and inhibitor composition of corn cob hydrolysate used in this study. (DOCX)

S3 Table List of primers used in this study. (DOCX)

## References

[1] Demeke MM, Dietz H, Li Y, Foulquie-Moreno MR, Mutturi S, Deprez S, et al. Development of a D-xylose fermenting and inhibitor tolerant industrial *Saccharomyces cerevisiae* strain with high performance in lignocellulose hydrolysates using metabolic and evolutionary engineering. Biotechnol Biofuels. 2013;6(1):89. Epub 2013/06/27. doi: 10.1186/1754-6834-6-89 ; PubMed Central PMCID: PMC3698012. 23800147 PMC3698012

[2] Bellissimi E, van Dijken JP, Pronk JT, van Maris AJ. Effects of acetic acid on the kinetics of xylose fermentation by an engineered, xylose-isomerase-based *Saccharomyces cerevisiae* strain. FEMS Yeast Res. 2009;9(3):358–64. Epub 2009/05/07. doi: 10.1111/j.1567-1364.2009.00487.x . 19416101

[3] Ask M, Bettiga M, Duraiswamy VR, Olsson L. Pulsed addition of HMF and furfural to batch-grown xylose-utilizing *Saccharomyces cerevisiae* results in different physiological responses in glucose and xylose consumption phase. Biotechnol Biofuels. 2013;6(1):181. Epub 2013/12/18. doi: 10.1186/1754-6834-6-181 ; PubMed Central PMCID: PMC3878631. 24341320 PMC3878631

[4] Mills TY, Sandoval NR, Gill RT. Cellulosic hydrolysate toxicity and tolerance mechanisms in *Escherichia coli*. Biotechnol Biofuels. 2009;2:26. Epub 2009/10/17. doi: 10.1186/1754-6834-2-26 ; PubMed Central PMCID: PMC2770041. 19832972 PMC2770041

[5] Kang Q, Appels L, Tan T, Dewil R. Bioethanol from lignocellulosic biomass: current findings determine research priorities. ScientificWorldJournal. 2014;2014:298153. Epub 2015/01/24. doi: 10.1155/2014/298153 ; PubMed Central PMCID: PMC4295598. 25614881 PMC4295598

[6] Caspeta L, Castillo T, Nielsen J. Modifying yeast tolerance to inhibitory conditions of ethanol production processes. Front Bioeng Biotechnol. 2015;3:184. Epub 2015/12/01. doi: 10.3389/fbioe.2015.00184 ; PubMed Central PMCID: PMC4641163. 26618154 PMC4641163

[7] Woo JM, Yang KM, Kim SU, Blank LM, Park JB. High temperature stimulates acetic acid accumulation and enhances the growth inhibition and ethanol production by *Saccharomyces cerevisiae* under fermenting conditions. Appl Microbiol Biotechnol. 2014;98(13):6085–94. Epub 2014/04/08. doi: 10.1007/s00253-014-5691-x . 24706214

[8] Jonsson LJ, Alriksson B, Nilvebrant NO. Bioconversion of lignocellulose: inhibitors and detoxification. Biotechnol Biofuels. 2013;6(1):16. Epub 2013/01/30. doi: 10.1186/1754-6834-6-16 ; PubMed Central PMCID: PMC3574029. 23356676 PMC3574029

[9] Allen SA, Clark W, McCaffery JM, Cai Z, Lanctot A, Slininger PJ, et al. Furfural induces reactive oxygen species accumulation and cellular damage in S*accharomyces cerevisiae*. Biotechnol Biofuels. 2010;3:2. Epub 2010/02/13. doi: 10.1186/1754-6834-3-2 ; PubMed Central PMCID: PMC2820483. 20150993 PMC2820483

[10] Janzowski C, Glaab V, Samimi E, Schlatter J, Eisenbrand G. 5-Hydroxymethylfurfural: assessment of mutagenicity, DNA-damaging potential and reactivity towards cellular glutathione. Food Chem Toxicol. 2000;38(9):801–9. Epub 2000/08/10. doi: 10.1016/s0278-6915(00)00070-3 . 10930701

[11] Modig T, Liden G, Taherzadeh MJ. Inhibition effects of furfural on alcohol dehydrogenase, aldehyde dehydrogenase and pyruvate dehydrogenase. Biochem J. 2002;363(Pt 3):769–76. Epub 2002/04/20. doi: 10.1042/0264-6021:3630769 ; PubMed Central PMCID: PMC1222530. 11964178 PMC1222530

[12] Petersson A, Almeida JR, Modig T, Karhumaa K, Hahn-Hagerdal B, Gorwa-Grauslund MF, et al. A 5-hydroxymethyl furfural reducing enzyme encoded by the *Saccharomyces cerevisiae ADH6* gene conveys HMF tolerance. Yeast. 2006;23(6):455–64. Epub 2006/05/03. doi: 10.1002/yea.1370 . 16652391

[13] Laadan B, Almeida JR, Radstrom P, Hahn-Hagerdal B, Gorwa-Grauslund M. Identification of an NADH-dependent 5-hydroxymethylfurfural-reducing alcohol dehydrogenase in *Saccharomyces cerevisiae*. Yeast. 2008;25(3):191–8. Epub 2008/02/28. doi: 10.1002/yea.1578 . 18302314

[14] Almeida JR, Roder A, Modig T, Laadan B, Liden G, Gorwa-Grauslund MF. NADH- vs NADPH-coupled reduction of 5-hydroxymethyl furfural (HMF) and its implications on product distribution in *Saccharomyces cerevisiae*. Appl Microbiol Biotechnol. 2008;78(6):939–45. Epub 2008/03/12. doi: 10.1007/s00253-008-1364-y . 18330568

[15] Park SE, Koo HM, Park YK, Park SM, Park JC, Lee OK, et al. Expression of aldehyde dehydrogenase 6 reduces inhibitory effect of furan derivatives on cell growth and ethanol production in *Saccharomyces cerevisiae*. Bioresour Technol. 2011;102(10):6033–8. Epub 2011/03/23. doi: 10.1016/j.biortech.2011.02.101 . 21421300

[16] Ishii J, Yoshimura K, Hasunuma T, Kondo A. Reduction of furan derivatives by overexpressing NADH-dependent Adh1 improves ethanol fermentation using xylose as sole carbon source with *Saccharomyces cerevisiae* harboring XR-XDH pathway. Appl Microbiol Biotechnol. 2013;97(6):2597–607. Epub 2012/09/25. doi: 10.1007/s00253-012-4376-6 . 23001007

[17] Liu ZL, Moon J. A novel NADPH-dependent aldehyde reductase gene from *Saccharomyces cerevisiae* NRRL Y-12632 involved in the detoxification of aldehyde inhibitors derived from lignocellulosic biomass conversion. Gene. 2009;446(1):1–10. Epub 2009/07/07. doi: 10.1016/j.gene.2009.06.018 . 19577617

[18] Liu ZL, Moon J, Andersh BJ, Slininger PJ, Weber S. Multiple gene-mediated NAD(P)H-dependent aldehyde reduction is a mechanism of in situ detoxification of furfural and 5-hydroxymethylfurfural by *Saccharomyces cerevisiae*. Appl Microbiol Biotechnol. 2008;81(4):743–53. Epub 2008/09/24. doi: 10.1007/s00253-008-1702-0 . 18810428

[19] Moon J, Liu ZL. Engineered NADH-dependent *GRE2* from *Saccharomyces cerevisiae* by directed enzyme evolution enhances HMF reduction using additional cofactor NADPH. Enzyme Microb Technol. 2012;50(2):115–20. Epub 2012/01/10. doi: 10.1016/j.enzmictec.2011.10.007 . 22226197

[20] Zhao X, Tang J, Wang X, Yang R, Zhang X, Gu Y, et al. *YNL134C* from *Saccharomyces cerevisiae* encodes a novel protein with aldehyde reductase activity for detoxification of furfural derived from lignocellulosic biomass. Yeast. 2015;32(5):409–22. Epub 2015/02/07. doi: 10.1002/yea.3068 . 25656244

[21] Wang H, Li Q, Zhang Z, Zhou C, Ayepa E, Abrha GT, et al. *YKL107W* from *Saccharomyces cerevisiae* encodes a novel aldehyde reductase for detoxification of acetaldehyde, glycolaldehyde, and furfural. Applied Microbiology and Biotechnology. 2019;103(14):5699–713. doi: 10.1007/s00253-019-09885-x 31115629

[22] Wang X, Liang Z, Hou J, Bao X, Shen Y. Identification and functional evaluation of the reductases and dehydrogenases from *Saccharomyces cerevisiae* involved in vanillin resistance. BMC Biotechnol. 2016;16:31. Epub 2016/04/03. doi: 10.1186/s12896-016-0264-y ; PubMed Central PMCID: PMC4818428. 27036139 PMC4818428

[23] Kim D, Hahn JS. Roles of the Yap1 transcription factor and antioxidants in *Saccharomyces cerevisiae*’s tolerance to furfural and 5-hydroxymethylfurfural, which function as thiol-reactive electrophiles generating oxidative stress. Appl Environ Microbiol. 2013;79(16):5069–77. Epub 2013/06/25. doi: 10.1128/AEM.00643-13 ; PubMed Central PMCID: PMC3754716. 23793623 PMC3754716

[24] Wallace-Salinas V, Signori L, Li YY, Ask M, Bettiga M, Porro D, et al. Re-assessment of *YAP1* and *MCR1* contributions to inhibitor tolerance in robust engineered *Saccharomyces cerevisiae* fermenting undetoxified lignocellulosic hydrolysate. AMB Express. 2014;4:56. Epub 2014/08/26. doi: 10.1186/s13568-014-0056-5 ; PubMed Central PMCID: PMC4105880. 25147754 PMC4105880

[25] Gorsich SW, Dien BS, Nichols NN, Slininger PJ, Liu ZL, Skory CD. Tolerance to furfural-induced stress is associated with pentose phosphate pathway genes *ZWF1*, *GND1*, *RPE1*, and *TKL1* in *Saccharomyces cerevisiae*. Appl Microbiol Biotechnol. 2006;71(3):339–49. Epub 2005/10/14. doi: 10.1007/s00253-005-0142-3 . 16222531

[26] Yee KL, Jansen LE, Lajoie CA, Penner MH, Morse L, Kelly CJ. Furfural and 5-hydroxymethyl-furfural degradation using recombinant manganese peroxidase. Enzyme Microb Technol. 2018;108:59–65. Epub 2017/11/08. doi: 10.1016/j.enzmictec.2017.08.009 . 29108628

[27] Laadan B, Wallace-Salinas V, Carlsson ÅJ, Almeida JRM, Rådström P, Gorwa-Grauslund MF. Furaldehyde substrate specificity and kinetics of *Saccharomyces cerevisiae* alcohol dehydrogenase 1 variants. Microbial Cell Factories. 2014;13(1):112. doi: 10.1186/s12934-014-0112-5 25287956 PMC4423641

[28] Cunha JT, Costa CE, Ferraz L, Romani A, Johansson B, Sa-Correia I, et al. *HAA1* and *PRS3* overexpression boosts yeast tolerance towards acetic acid improving xylose or glucose consumption: unravelling the underlying mechanisms. Appl Microbiol Biotechnol. 2018;102(10):4589–600. Epub 2018/04/03. doi: 10.1007/s00253-018-8955-z . 29607452

[29] Swinnen S, Henriques SF, Shrestha R, Ho P-W, Sá-Correia I, Nevoigt E. Improvement of yeast tolerance to acetic acid through Haa1 transcription factor engineering: towards the underlying mechanisms. Microbial Cell Factories. 2017;16(1):7. doi: 10.1186/s12934-016-0621-5 28068993 PMC5220606

[30] Tanaka K, Ishii Y, Ogawa J, Shima J. Enhancement of acetic acid tolerance in *Saccharomyces cerevisiae* by overexpression of the *HAA1* gene, encoding a transcriptional activator. Appl Environ Microbiol. 2012;78(22):8161–3. Epub 2012/09/11. doi: 10.1128/AEM.02356-12 ; PubMed Central PMCID: PMC3485965. 22961896 PMC3485965

[31] Almario MP, Reyes LH, Kao KC. Evolutionary engineering of *Saccharomyces cerevisiae* for enhanced tolerance to hydrolysates of lignocellulosic biomass. Biotechnol Bioeng. 2013;110(10):2616–23. Epub 2013/04/25. doi: 10.1002/bit.24938 . 23613173

[32] Koppram R, Albers E, Olsson L. Evolutionary engineering strategies to enhance tolerance of xylose utilizing recombinant yeast to inhibitors derived from spruce biomass. Biotechnol Biofuels. 2012;5(1):32. Epub 2012/05/15. doi: 10.1186/1754-6834-5-32 ; PubMed Central PMCID: PMC3408370. 22578262 PMC3408370

[33] Billal DS, Feng J, Leprohon P, Légaré D, Ouellette M. Whole genome analysis of linezolid resistance in *Streptococcus pneumoniae* reveals resistance and compensatory mutations. BMC Genomics. 2011;12:512. Epub 2011/10/19. doi: 10.1186/1471-2164-12-512 ; PubMed Central PMCID: PMC3212830. 22004526 PMC3212830

[34] Fani F, Leprohon P, Zhanel GG, Bergeron MG, Ouellette M. Genomic analyses of DNA transformation and penicillin resistance in *Streptococcus pneumoniae* clinical isolates. Antimicrob Agents Chemother. 2014;58(3):1397–403. Epub 2013/12/18. doi: 10.1128/AAC.01311-13 ; PubMed Central PMCID: PMC3957846. 24342643 PMC3957846

[35] Mell JC, Hall IM, Redfield RJ. Defining the DNA uptake specificity of naturally competent *Haemophilus influenzae* cells. Nucleic Acids Research. 2012;40(17):8536–49. doi: 10.1093/nar/gks640 22753031 PMC3458573

[36] Ishida-Fujii K, Goto S, Sugiyama H, Takagi Y, Saiki T, Takagi M. Breeding of flocculent industrial alcohol yeast strains by self-cloning of the flocculation gene FLO1 and repeated-batch fermentation by transformants. J Gen Appl Microbiol. 1998;44(5):347–53. Epub 2002/12/27. doi: 10.2323/jgam.44.347 . 12501414

[37] Zhang W, Geng A. Improved ethanol production by a xylose-fermenting recombinant yeast strain constructed through a modified genome shuffling method. Biotechnology for Biofuels. 2012;5(1):46. doi: 10.1186/1754-6834-5-46 22809265 PMC3463424

[38] Stojiljkovic M, Foulquié-Moreno MR, Thevelein JM. Polygenic analysis of very high acetic acid tolerance in the yeast *Saccharomyces cerevisiae* reveals a complex genetic background and several new causative alleles. Biotechnology for Biofuels. 2020;13(1):126. doi: 10.1186/s13068-020-01761-5 32695222 PMC7364526

[39] Deparis Q, Duitama J, Foulquie-Moreno MR, Thevelein JM. Whole-genome transformation promotes tRNA anticodon suppressor mutations under stress. mBio. 2021;12(2). Epub 2021/03/25. doi: 10.1128/mBio.03649-20 ; PubMed Central PMCID: PMC8092322. 33758086 PMC8092322

[40] Avrahami-Moyal L, Engelberg D, Wenger JW, Sherlock G, Braun S. Turbidostat culture of *Saccharomyces cerevisiae* W303-1A under selective pressure elicited by ethanol selects for mutations in *SSD1* and *UTH1*. FEMS Yeast Res. 2012;12(5):521–33. Epub 2012/03/27. doi: 10.1111/j.1567-1364.2012.00803.x ; PubMed Central PMCID: PMC3393845. 22443114 PMC3393845

[41] Wahlbom CF, van Zyl WH, Jonsson LJ, Hahn-Hagerdal B, Otero RR. Generation of the improved recombinant xylose-utilizing *Saccharomyces cerevisiae* TMB 3400 by random mutagenesis and physiological comparison with *Pichia stipitis* CBS 6054. FEMS Yeast Res. 2003;3(3):319–26. Epub 2003/04/12. doi: 10.1016/S1567-1356(02)00206-4 . 12689639

[42] Linden T, Peetre J, Hahn-Hagerdal B. Isolation and characterization of acetic acid-tolerant galactose-fermenting strains of *Saccharomyces cerevisiae* from a spent sulfite liquor fermentation plant. Appl Environ Microbiol. 1992;58(5):1661–9. Epub 1992/05/01. doi: 10.1128/aem.58.5.1661-1669.1992 ; PubMed Central PMCID: PMC195655. 1622236 PMC195655

[43] Nilsson A, Gorwa-Grauslund MF, Hahn-Hagerdal B, Liden G. Cofactor dependence in furan reduction by *Saccharomyces cerevisiae* in fermentation of acid-hydrolyzed lignocellulose. Appl Environ Microbiol. 2005;71(12):7866–71. Epub 2005/12/08. doi: 10.1128/AEM.71.12.7866-7871.2005 ; PubMed Central PMCID: PMC1317483. 16332761 PMC1317483

[44] Peter J, De Chiara M, Friedrich A, Yue JX, Pflieger D, Bergstrom A, et al. Genome evolution across 1,011 *Saccharomyces cerevisiae* isolates. Nature. 2018;556(7701):339–44. Epub 2018/04/13. doi: 10.1038/s41586-018-0030-5 ; PubMed Central PMCID: PMC6784862. 29643504 PMC6784862

[45] Lee WG, Lee JS, Shin CS, Park SC, Chang HN, Chang YK. Ethanol production using concentrated oak wood hydrolysates and methods to detoxify. Appl Biochem Biotechnol. 1999;77–79:547–59. Epub 2004/08/12. doi: 10.1385/abab:78:1-3:547 . 15304722

[46] Zha Y, Westerhuis JA, Muilwijk B, Overkamp KM, Nijmeijer BM, Coulier L, et al. Identifying inhibitory compounds in lignocellulosic biomass hydrolysates using an exometabolomics approach. BMC Biotechnol. 2014;14:22. Epub 2014/03/25. doi: 10.1186/1472-6750-14-22 ; PubMed Central PMCID: PMC3998114. 24655423 PMC3998114

[47] Heer D, Sauer U. Identification of furfural as a key toxin in lignocellulosic hydrolysates and evolution of a tolerant yeast strain. Microb Biotechnol. 2008;1(6):497–506. Epub 2008/11/01. doi: 10.1111/j.1751-7915.2008.00050.x ; PubMed Central PMCID: PMC3815291. 21261870 PMC3815291

[48] Mandenius CF, Lid n H, Eklov T, Taherzadeh MJ, Liden G. Predicting fermentability of wood hydrolyzates with responses from electronic noses. Biotechnol Prog. 1999;15(4):617–21. Epub 1999/08/12. doi: 10.1021/bp990059d . 10441352

[49] Taherzadeh MJ, Eklund R, Gustafsson L, Niklasson C, Lidén G. Characterization and fermentation of dilute-acid hydrolyzates from wood. Industrial & Engineering Chemistry Research. 1997;36(11):4659–65. doi: 10.1021/ie9700831

[50] Field SJ, Ryden P, Wilson D, James SA, Roberts IN, Richardson DJ, et al. Identification of furfural resistant strains of *Saccharomyces cerevisiae* and *Saccharomyces paradoxus* from a collection of environmental and industrial isolates. Biotechnol Biofuels. 2015;8:33. Epub 2015/04/11. doi: 10.1186/s13068-015-0217-z ; PubMed Central PMCID: PMC4389715. 25861389 PMC4389715

[51] Larsson S, Palmqvist E, Hahn-Hägerdal B, Tengborg C, Stenberg K, Zacchi G, et al. The generation of fermentation inhibitors during dilute acid hydrolysis of softwood. Enzyme and Microbial Technology. 1999;24(3):151–9. 10.1016/S0141-0229(98)00101-X.

[52] Cocchi M, Ferrari G, Manzini D, Marchetti A, Sighinolfi S. Study of the monosaccharides and furfurals evolution during the preparation of cooked grape musts for Aceto Balsamico Tradizionale production. Journal of Food Engineering. 2007;79(4):1438–44. 10.1016/j.jfoodeng.2006.01.091.

[53] Tagliazucchi D, Verzelloni E, Helal A, Conte A. Effect of grape variety on the evolution of sugars, hydroxymethylfurfural, polyphenols and antioxidant activity during grape must cooking. International Journal of Food Science & Technology. 2013;48(4):808–16. doi: 10.1111/ijfs.12031

[54] Moller HD, Parsons L, Jorgensen TS, Botstein D, Regenberg B. Extrachromosomal circular DNA is common in yeast. Proc Natl Acad Sci U S A. 2015;112(24):E3114–22. Epub 2015/06/04. doi: 10.1073/pnas.1508825112 ; PubMed Central PMCID: PMC4475933. 26038577 PMC4475933

[55] Iparraguirre L, Prada-Luengo I, Regenberg B, Otaegui D. To be or not to be: circular RNAs or mRNAs from circular DNAs? Front Genet. 2019;10:940. Epub 2019/11/05. doi: 10.3389/fgene.2019.00940 ; PubMed Central PMCID: PMC6797608. 31681407 PMC6797608

[56] Riveros-Rosas H, Julian-Sanchez A, Villalobos-Molina R, Pardo JP, Pina E. Diversity, taxonomy and evolution of medium-chain dehydrogenase/reductase superfamily. Eur J Biochem. 2003;270(16):3309–34. Epub 2003/08/06. doi: 10.1046/j.1432-1033.2003.03704.x . 12899689

[57] Chang A, Fink GR. Targeting of the yeast plasma membrane [H+]ATPase: a novel gene *AST1* prevents mislocalization of mutant ATPase to the vacuole. J Cell Biol. 1995;128(1–2):39–49. Epub 1995/01/01. doi: 10.1083/jcb.128.1.39 ; PubMed Central PMCID: PMC2120329. 7822420 PMC2120329

[58] Bagnat M, Chang A, Simons K. Plasma membrane proton ATPase Pma1p requires raft association for surface delivery in yeast. Mol Biol Cell. 2001;12(12):4129–38. Epub 2001/12/12. doi: 10.1091/mbc.12.12.4129 ; PubMed Central PMCID: PMC60781. 11739806 PMC60781

[59] Heer D, Heine D, Sauer U. Resistance of *Saccharomyces cerevisiae* to high concentrations of furfural is based on NADPH-dependent reduction by at least two oxireductases. Appl Environ Microbiol. 2009;75(24):7631–8. Epub 2009/10/27. doi: 10.1128/AEM.01649-09 ; PubMed Central PMCID: PMC2794096. 19854918 PMC2794096

[60] Sehnem NT, Machado Ada S, Leite FC, Pita Wde B, de Morais MA Jr., Ayub MA. 5-Hydroxymethylfurfural induces *ADH7* and *ARI1* expression in tolerant industrial *Saccharomyces cerevisiae* strain P6H9 during bioethanol production. Bioresour Technol. 2013;133:190–6. Epub 2013/02/21. doi: 10.1016/j.biortech.2013.01.063 . 23422309

[61] Liu ZL, Slininger PJ, Gorsich SW. Enhanced biotransformation of furfural and hydroxymethylfurfural by newly developed ethanologenic yeast strains. Appl Biochem Biotechnol. 121–124. 2005/05/27 ed2005. p. 451–60. 15917621

[62] Keating JD, Panganiban C, Mansfield SD. Tolerance and adaptation of ethanologenic yeasts to lignocellulosic inhibitory compounds. Biotechnol Bioeng. 2006;93(6):1196–206. Epub 2006/02/14. doi: 10.1002/bit.20838 . 16470880

[63] Sanchez INV, Bettiga M, Gorwa-Grauslund MF. Isolation and characterization of a resident tolerant *Saccharomyces cerevisiae* strain from a spent sulfite liquor fermentation plant. AMB Express. 2012;2(1):68. Epub 2012/12/15. doi: 10.1186/2191-0855-2-68 ; PubMed Central PMCID: PMC3539867. 23237549 PMC3539867

[64] Shen Y, Li H, Wang X, Zhang X, Hou J, Wang L, et al. High vanillin tolerance of an evolved *Saccharomyces cerevisiae* strain owing to its enhanced vanillin reduction and antioxidative capacity. J Ind Microbiol Biotechnol. 2014;41(11):1637–45. Epub 2014/09/30. doi: 10.1007/s10295-014-1515-3 . 25261986

[65] Wang X, Gao Q, Bao J. Enhancement of furan aldehydes conversion in *Zymomonas mobilis* by elevating dehydrogenase activity and cofactor regeneration. Biotechnol Biofuels. 2017;10:24. Epub 2017/02/07. doi: 10.1186/s13068-017-0714-3 ; PubMed Central PMCID: PMC5282692. 28163781 PMC5282692

[66] Almeida JR, Modig T, Roder A, Liden G, Gorwa-Grauslund MF. *Pichia stipitis* xylose reductase helps detoxifying lignocellulosic hydrolysate by reducing 5-hydroxymethyl-furfural (HMF). Biotechnol Biofuels. 2008;1(1):12. Epub 2008/06/13. doi: 10.1186/1754-6834-1-12 ; PubMed Central PMCID: PMC2464581. 18547412 PMC2464581

[67] Miller EN, Jarboe LR, Yomano LP, York SW, Shanmugam KT, Ingram LO. Silencing of NADPH-dependent oxidoreductase genes (*yqhD* and *dkgA*) in furfural-resistant ethanologenic *Escherichia coli*. Appl Environ Microbiol. 2009;75(13):4315–23. Epub 2009/05/12. doi: 10.1128/AEM.00567-09 ; PubMed Central PMCID: PMC2704836. 19429550 PMC2704836

[68] Palmqvist E, Almeida JS, Hahn-Hagerdal B. Influence of furfural on anaerobic glycolytic kinetics of *Saccharomyces cerevisiae* in batch culture. Biotechnol Bioeng. 1999;62(4):447–54. Epub 1999/01/28. doi: 10.1002/(sici)1097-0290(19990220)62:4&lt;447::aid-bit7&gt;3.0.co;2-0 . 9921153

[69] Sarvari Horvath I, Franzen CJ, Taherzadeh MJ, Niklasson C, Liden G. Effects of furfural on the respiratory metabolism of *Saccharomyces cerevisiae* in glucose-limited chemostats. Appl Environ Microbiol. 2003;69(7):4076–86. Epub 2003/07/04. doi: 10.1128/AEM.69.7.4076-4086.2003 ; PubMed Central PMCID: PMC165176. 12839784 PMC165176

[70] Wahlbom CF, Hahn-Hagerdal B. Furfural, 5-hydroxymethyl furfural, and acetoin act as external electron acceptors during anaerobic fermentation of xylose in recombinant *Saccharomyces cerevisiae*. Biotechnol Bioeng. 2002;78(2):172–8. Epub 2002/03/01. doi: 10.1002/bit.10188 . 11870608

[71] Stanley GA, Douglas NG, Every EJ, Tzanatos T, Pamment NB. Inhibition and stimulation of yeast growth by acetaldehyde. Biotechnology Letters. 1993;15:1199–204.

[72] Mukherjee V, Radecka D, Aerts G, Verstrepen KJ, Lievens B, Thevelein JM. Phenotypic landscape of non-conventional yeast species for different stress tolerance traits desirable in bioethanol fermentation. Biotechnol Biofuels. 2017;10:216. Epub 2017/09/20. doi: 10.1186/s13068-017-0899-5 ; PubMed Central PMCID: PMC5597992. 28924451 PMC5597992

[73] Benatuil L, Perez JM, Belk J, Hsieh CM. An improved yeast transformation method for the generation of very large human antibody libraries. Protein Eng Des Sel. 2010;23(4):155–9. Epub 2010/02/05. doi: 10.1093/protein/gzq002 . 20130105

[74] Gietz RD, Schiestl RH. High-efficiency yeast transformation using the LiAc/SS carrier DNA/PEG method. Nat Protoc. 2007;2(1):31–4. Epub 2007/04/03. doi: 10.1038/nprot.2007.13 . 17401334

[75] Duitama J, Sanchez-Rodriguez A, Goovaerts A, Pulido-Tamayo S, Hubmann G, Foulquie-Moreno MR, et al. Improved linkage analysis of Quantitative Trait Loci using bulk segregants unveils a novel determinant of high ethanol tolerance in yeast. BMC Genomics. 2014;15:207. Epub 2014/03/20. doi: 10.1186/1471-2164-15-207 ; PubMed Central PMCID: PMC4003806. 24640961 PMC4003806

[76] Langmead B, Salzberg SL. Fast gapped-read alignment with Bowtie 2. Nat Methods. 2012;9(4):357–9. Epub 2012/03/06. doi: 10.1038/nmeth.1923 ; PubMed Central PMCID: PMC3322381. 22388286 PMC3322381

[77] Benson G. Tandem repeats finder: a program to analyze DNA sequences. Nucleic Acids Res. 1999;27(2):573–80. Epub 1998/12/24. doi: 10.1093/nar/27.2.573 ; PubMed Central PMCID: PMC148217. 9862982 PMC148217

[78] Mali P, Yang L, Esvelt KM, Aach J, Guell M, DiCarlo JE, et al. RNA-guided human genome engineering via Cas9. Science. 2013;339(6121):823–6. Epub 2013/01/05. doi: 10.1126/science.1232033 ; PubMed Central PMCID: PMC3712628. 23287722 PMC3712628

[79] Li X. RbCl super competent cells. Bio-protocol. 2011;1(11):e76. doi: 10.21769/BioProtoc.76

