# Supplementary figures and images for: A novel AST2 mutation generated upon whole-genome transformation of Saccharomyces cerevisiae confers high tolerance to 5-Hydroxymethylfurfural (HMF) and other inhibitors
Source: PLoS Genet. 2021 Oct 8;17(10):e1009826. doi: 10.1371/journal.pgen.1009826 (PMC8500407; doi:10.1371/journal.pgen.1009826)

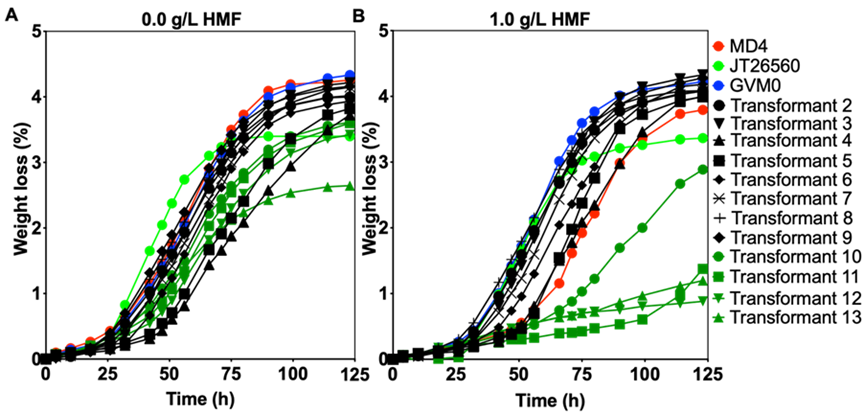

Supplement: S1 Fig — Evaluation of the fermentation capacity of WG transformants in the presence of HMF in small-scale fermentations (10 mL) in corn cob hydrolysate enriched with (A) 0.0 g/L or (B) 1.0 g/L HMF, pH 5.2, 35°C, 350 rpm and initial OD 5.0. The GVM0 strain and Transformants 2 to 9 were obtained by transformation of strain MD4 with gDNA of C. glabrata strain JT26560, Transformant 10 with gDNA from wine yeast DBVPG 1552 (JT25869), Transformant 11 with own gDNA of MD4, Transformant 12 with water, and Transformant 13 with gDNA from lab strain S288c. The three latter conditions never resulted in strains with stable improved HMF tolerance, neither when grown on nutrient plates nor when evaluated in small-scale fermentations. Three biological replicates were performed for the strains MD4, JT26560 and GVM0. Two technical replicates were performed for strain MD4. All other strains were evaluated once. (TIFF) [file pgen.1009826.s001.tiff]

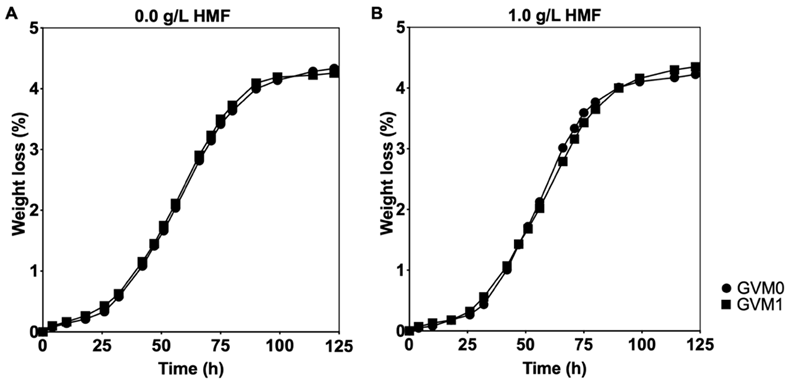

Supplement: S2 Fig — Small-scale fermentations (10 mL) were performed in corn cob hydrolysate enriched with 0.0 g/L or 1.0 g/L HMF, pH 5.2, 35°C, 350 rpm and initial OD 5.0. Representative result of two biological replicates is shown. (TIFF) [file pgen.1009826.s002.tiff]

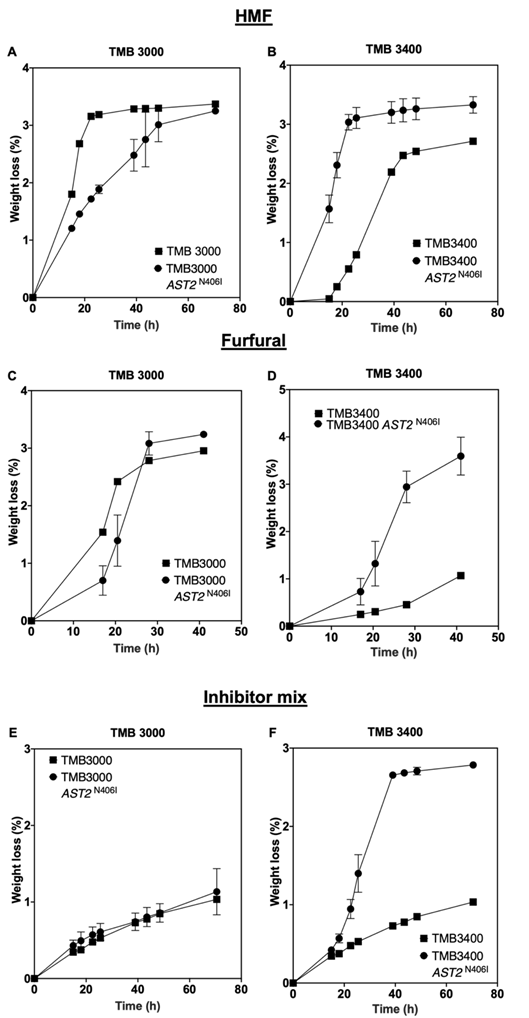

Supplement: S4 Fig — Small-scale fermentations (10 mL) were performed at 35°C, 350 rpm, initial OD 5.0 in YPDX, enriched with 12.0 g/L HMF at pH 5.2 (A, B), 4.0 g/L furfural at pH 5.2 (C, D) and an inhibitor mixture of 2.80 g/L HMF, 1.75 g/L furfural, 0.35 g/L vanillin and 4.20 g/L acetic acid at pH 4.6 (E, F). Mean values with standard deviation are shown for three independent transformants of strains TMB 3000 and TMB 3400, or two technical replicates for strains TMB 3000 and TMB 3400. The experiment was performed once. (TIFF) [file pgen.1009826.s004.tiff]

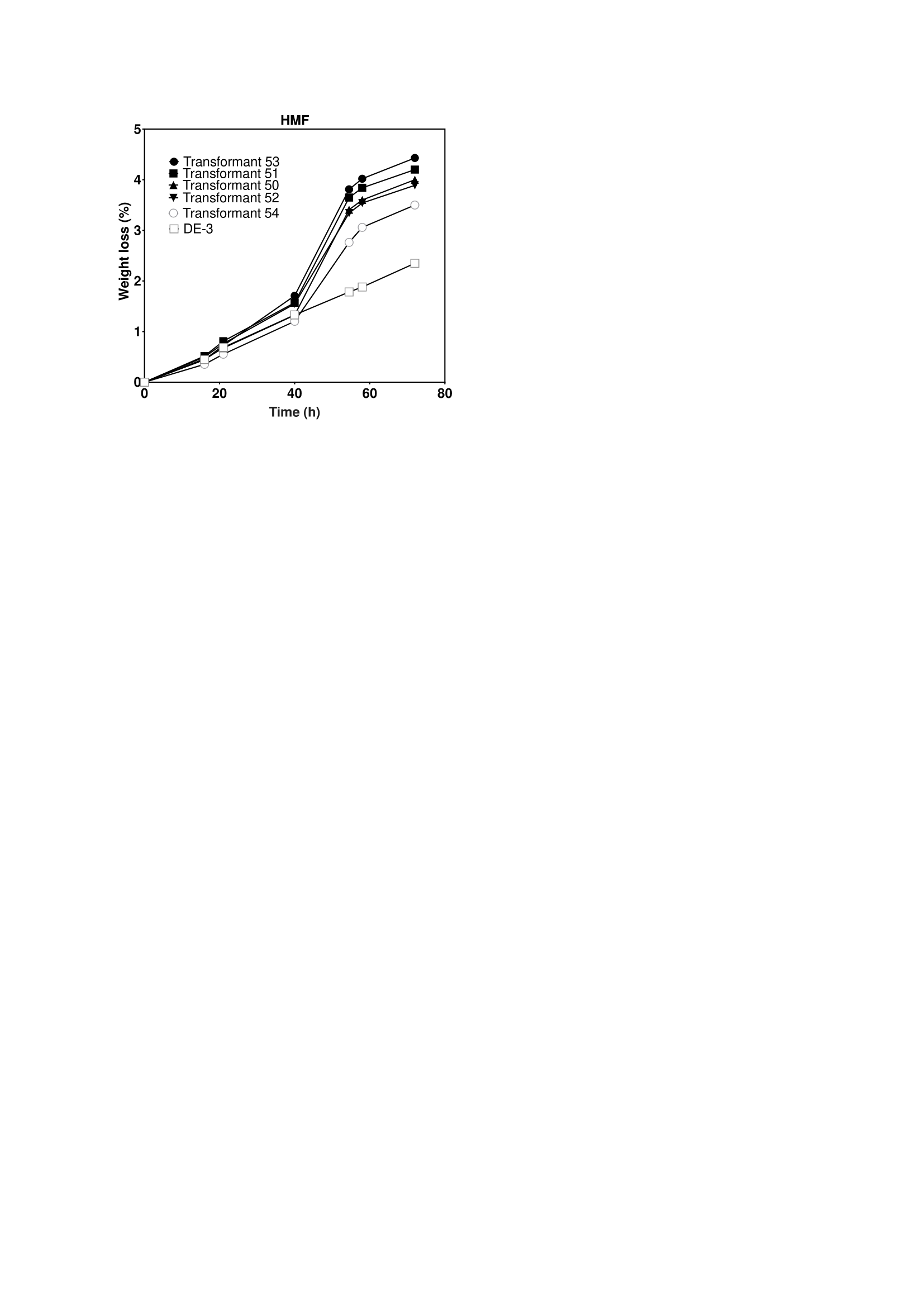

Supplement: S5 Fig — Evaluation of the fermentation performance of WG transformants of DE-3 (i.e. Transformant 50, 51, 52, 53, 54), selected for improved HMF tolerance, in small-scale fermentations (10 mL, pH 5.2, 35°C, initial OD 5.0, 350 rpm in synthetic YPDX medium enriched with 12 g/L HMF). The strains were evaluated once except for strain DE-3 for which two technical replicates were used. The experiment was performed once. (TIFF) [file pgen.1009826.s005.tiff]

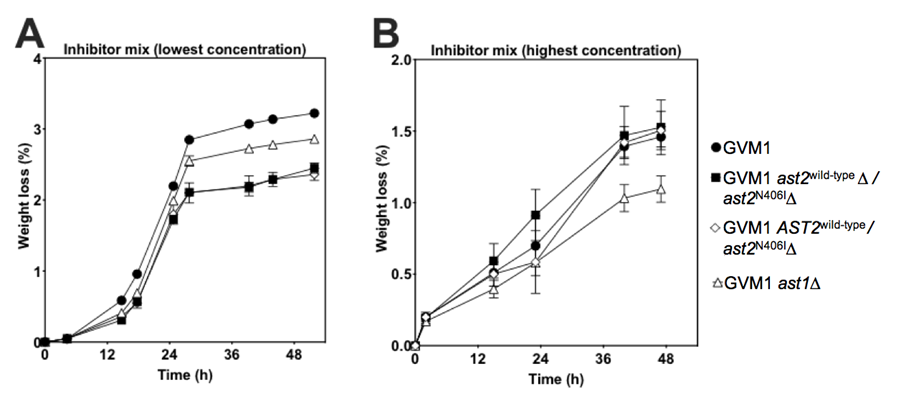

Supplement: S6 Fig — Small-scale fermentations (10 mL) were performed at pH 4.6, 35°C, 350 rpm, initial OD600 of 5.0 in YPDX with a mixture of (A) 2.80 g/L HMF, 1.75 g/L furfural, 0.35 g/L vanillin and 4.20 g/L acetic acid, or (B) 3.36 g/L HMF, 2.10 g/L furfural, 0.42 g/L vanillin and 5.04 g/L acetic acid. Mean values with standard deviation are shown for three independent transformants of the derivatives of GVM1, or three technical replicates for the strain GVM1. The experiment was performed once. (TIFF) [file pgen.1009826.s006.tiff]

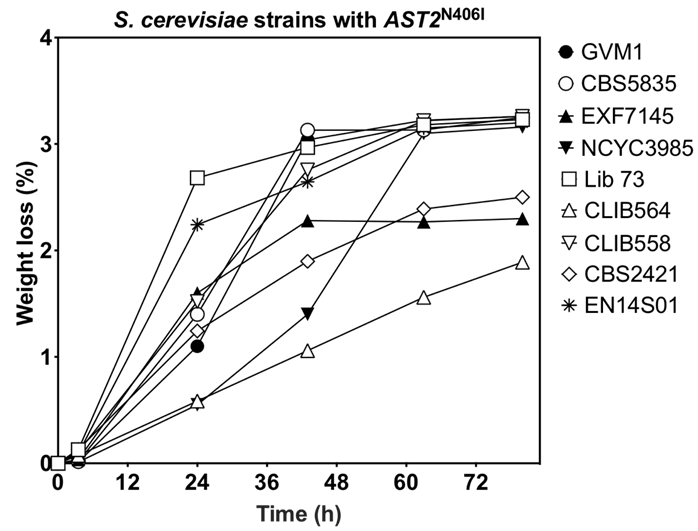

Supplement: S7 Fig — Small-scale fermentations (10 mL) were performed in YPDX medium enriched with 12.0 g/L HMF, pH 5.2, 35°C, 350 rpm and initial OD 5.0. Strains depicted are GVM1, a wine yeast (CBS5835​), a natural isolate from oak (EXF7145), a natural isolate from wax on rock surface (NCYC3985), an isolate from grape must (Lib 73), two isolates from dairy cheese camembert (CLIB564 and CLIB558), an isolate from Japanese kefyr grains (CBS2421) and a soil isolate from Taiwan (EN14S01). The strains were evaluated once except for strain GVM1 for which two technical replicates were used. The experiment was performed once. (TIFF) [file pgen.1009826.s007.tiff]
