# Supplementary material for: A novel AST2 mutation generated upon whole-genome transformation of Saccharomyces cerevisiae confers high tolerance to 5-Hydroxymethylfurfural (HMF) and other inhibitors
Source: PLoS Genet. 2021 Oct 8;17(10):e1009826. doi: 10.1371/journal.pgen.1009826 (PMC8500407; doi:10.1371/journal.pgen.1009826)
Supplement: S1 Table — (DOCX) [file pgen.1009826.s008.docx]

**S1 Table. List of yeast strains used in this study**

| **Yeast strain** | **Description** | **Source and/or reference** |
| --- | --- | --- |
| MD4 | 2G yeast strain | Own collection |
| T18 | 2G yeast strain | Own collection |
| MD104 | 2G yeast strain | Own collection |
| JT26560 | HMF tolerant *Candida glabrata* strain obtained from grape must | Own collection |
| FY 431 | a *Saccharomyces boulardii* strain | Own collection |
| FY 550 | a *Pichia kluyverii* isolated from a vineyard | Own collection |
| FY 609 | a *Kluyveromyces marxianus* *CBS6499* | Own collection |
| FY 593 | a *Kluyveromyces marxianus* *CBS2080* | Own collection |
| FY 546 | a *Saccharomyces servazii* strain isolated from oak | Own collection |
| FY 548 | a *Saccharomyces servazii* strain isolated from oak | Own collection |
| FY 549 | a *Saccharomyces servazii* strain isolated from oak | Own collection |
| JT25869 | HMF tolerant wine strain | Own collection |
| JT22221 | HMF and furfural tolerant Anchor baker’s yeast strain | Own collection |
| JT23163 | HMF tolerant Merlot wine strain | Own collection |
| JT23339 | Zymaflore X16 wine strain | Own collection |
| JT23151 | HMF and furfural tolerant Fermicru 4F9 wine strain | Own collection |
| VR1 | HMF tolerant strain isolated at Santelisa Vale Bioenergia S/A – Morro Agudo – Sao Paulo, Brazil | Own collection |
| JT23156 | HMF and furfural tolerant Fermi PDM wine strain | Own collection |
| JT23341 | HMF tolerant Zymaflore VL3 wine strain | Own collection |
| JT22232 | ICV D47 multi-inhibitor tolerant wine strain | Own collection |
| JT23146 | HMF and furfural tolerant VIN7 wine strain | Own collection |
| JT21620 | HMF tolerant gold star baker’s yeast strain | Own collection |
| JT21653 | HMF tolerant Fermipan baker’s yeast strain | Own collection |
| FY 431 | HMF and furfural tolerant Sb. P strain | Own collection |
| CEN.Pk1 | Lab strain | Own collection |
| S288C | Non-HMF tolerant lab strain | Own collection |
| JT25416 | Levulinic acid tolerant, non-HMF tolerant strain isolated from grape must | Own collection |
| JT25880 | Formic acid tolerant, non-HMF tolerant strain isolated from grape must | Own collection |
| JT22277 | EXF, weak acid tolerant, non-HMF tolerant strain isolated from vinegar | Own collection |
| JT22689 | PYCC4542, weak acid tolerant, non-HMF tolerant strain isolated from fermentation must | Own collection |
| GVM0 | WG Transformant of MD4 with gDNA of JT26560 | This study |
| Transformant 2->9 | WG Transformants of MD4 with gDNA of JT26560 | This study |
| Transformant 10 | WG Transformant of MD4 with gDNA of JT25869 | This study |
| Transformant 11 | WG Transformant of MD4 with gDNA of MD4 | This study |
| Transformant 12 | WG Transformant of MD4 transformed with no gDNA | This study |
| Transformant 13 | WG Transformant of MD4 with gDNA of S288C | This study |
| GVM1 | Diploid segregant of GVM0 | This study |
| GVM1 REG2 mutant | GVM1 with deletion of wild type allele of *REG2* | This study |
| GVM1 REG2 wild-type | GVM1 with deletion of mutant allele of *REG2* | This study |
| GVM1 SAS3 mutant | GVM1 with deletion of wild type allele of *SAS3* | This study |
| GVM1 SAS3 wild-type | GVM1 with deletion of mutant allele of *SAS3* | This study |
| GVM1 DPP1 mutant | GVM1 with deletion of wild type allele of *DPP1* | This study |
| GVM1 DPP1 wild-type | GVM1 with deletion of mutant allele of *DPP1* | This study |
| GVM1 GIC2 mutant | GVM1 with deletion of wild type allele of *GIC2* | This study |
| GVM1 GIC2 wild-type | GVM1 with deletion of mutant allele of *GIC2* | This study |
| GVM1 AST2 mutant | GVM1 with deletion of wild type allele of *AST2* | This study |
| GVM1 AST2 wild-type | GVM1 with deletion of mutant allele of *AST2* | This study |
| GVM1 IES1 mutant | GVM1 with deletion of wild type allele of *IES1* | This study |
| GVM1 IES1 wild-type | GVM1 with deletion of mutant allele of *IES1* | This study |
| GVM1 ASG1 mutant | GVM1 with deletion of wild type allele of *ASG1* | This study |
| GVM1 ASG1 wild-type | GVM1 with deletion of mutant allele of *ASG1* | This study |
| GVM1 SYC1 mutant | GVM1 with deletion of wild type allele of *SYC1* | This study |
| GVM1 SYC1 wild-type | GVM1 with deletion of mutant allele of *SYC1* | This study |
| GVM1 TAH18 mutant | GVM1 with deletion of wild type allele of *TAH18* | This study |
| GVM1 TAH18 wild-type | GVM1 with deletion of mutant allele of *TAH18* | This study |
| GVM1 GDH3 mutant | GVM1 with deletion of wild type allele of *GDH3* | This study |
| GVM1 GDH3 wild-type | GVM1 with deletion of mutant allele of *GDH3* | This study |
| GVM1 YGL185C mutant | GVM1 with deletion of wild type allele of *YGL185C* | This study |
| GVM1 YGL185C wild-type | GVM1 with deletion of mutant allele of *YGL185C* | This study |
| GVM1 HXT2 mutant | GVM1 with deletion of wild type allele of *HXT2* | This study |
| GVM1 HXT2 wild-type | GVM1 with deletion of mutant allele of *HXT2* | This study |
| GVM1 FAS2 mutant | GVM1 with deletion of wild type allele of *FAS2* | This study |
| GVM1 FAS2 wild-type | GVM1 with deletion of mutant allele of *FAS2* | This study |
| GVM1 HSP82 mutant | GVM1 with deletion of wild type allele of *HSP82* | This study |
| GVM1 HSP82 wild-type | GVM1 with deletion of mutant allele of *HSP82* | This study |
| Segregant 1 - 8 | Segregants of GVM1 | This study |
| MD4.1 | MD4 engineered with 1 copy of *AST2*^N406I^ | This study |
| MD4.4 | MD4 engineered with 4 copies of *AST2*^N406I^ | This study |
| TMB3400 | 2G-bioethanols train | (1) |
| TMB3400-AST2 | TMB3400 engineered with *AST2*^N406I^ | This study |
| TMB 3000 | Low ethyl acetate producing strain, isolated from a paper sludge fermentation plant in Sweden. Diploid | (2) |
| TMB3000-AST2 | TMB3000 *AST2*^N406I^ | This study |
| CBS5835 | Wine, Spain  *S. cerevisiae* strain containing *AST2^406I^* SNP | (3) |
| EXF7145 | Oak (Quercus), Serbia  *S. cerevisiae* strain containing *AST2^406I^* SNP | (3) |
| NCYC3985 | Wax on rock surface, Bolivar, Ecuador  *S. cerevisiae* strain containing *AST2^406I^* SNP | (3) |
| CHF (Lib 73) | Grape must, Lebanon  *S. cerevisiae* strain containing *AST2^406I^* SNP | (3) |
| BGR (CLIB564) | Camembert, Normandy, France  *S. cerevisiae* strain containing *AST2^406I^* SNP | (3) |
| BGK (CLIB558) | Camembert, Normandy, France  *S. cerevisiae* strain containing *AST2^406I^* SNP | (3) |
| ARS (CBS2421) | Japanese kefyr grains  *S. cerevisiae* strain containing *AST2^406I^* SNP | (3) |
| AMH (EN14S01) | Soil, Sinyi, Nantou, Taiwan  *S. cerevisiae* strain containing *AST2^406I^* SNP | (3) |
| MD4.1 | MD4 engineered with 1 copy of *AST2*^N406I^ | This study |
| MD4.4 | Transformants of MD4 engineered with 4 copies of *AST2*^N406I^ | This study |
| GVM50 | Ethanol Red engineered with *AST2*^N406I^ | This study |
| GVM1 *AST1^D405I^* | GVM1 engineered with 2 copies of *AST1^D405I^* | This study |
| MD4 *AST1^D405I^* | MD4 engineered with 4 copies of *AST1^D405I^* | This study |
| GVM1 ΔΔ*AST2* | GVM1 double deletion strain of *AST2*^N406I^ and *AST2* wild-type | This study |
| GVM1 Δ*AST1* | GVM1 single deletion strain of *AST1* | This study |

**References**

1. Wahlbom CF, van Zyl WH, Jonsson LJ, Hahn-Hagerdal B, & Otero RR (2003) Generation of the improved recombinant xylose-utilizing *Saccharomyces cerevisiae* TMB 3400 by random mutagenesis and physiological comparison with *Pichia stipitis* CBS 6054. *FEMS Yeast Res* 3:319-326.

2. Linden T, Peetre J, & Hahn-Hagerdal B (1992) Isolation and characterization of acetic acid-tolerant galactose-fermenting strains of *Saccharomyces cerevisiae* from a spent sulfite liquor fermentation plant. *Appl Environ Microbiol* 58:1661-1669.

3. Peter J*, et al.* (2018) Genome evolution across 1,011 *Saccharomyces cerevisiae* isolates. *Nature* 556:339-344.
