# Supplementary material for: A novel AST2 mutation generated upon whole-genome transformation of Saccharomyces cerevisiae confers high tolerance to 5-Hydroxymethylfurfural (HMF) and other inhibitors
Source: PLoS Genet. 2021 Oct 8;17(10):e1009826. doi: 10.1371/journal.pgen.1009826 (PMC8500407; doi:10.1371/journal.pgen.1009826)
Supplement: S2 Table — (DOCX) [file pgen.1009826.s009.docx]

**S2 Table. Sugar and inhibitor composition of corn cob hydrolysate used in this study**

| **Hydrolysate**  **Component** | **Corn Cob** |
| --- | --- |
| Glucose | 6.88% |
| Xylose | 5.66% |
| Arabinose | 0.47% |
| Acetic Acid | 0.63% |
| Levulinic Acid | 0.00% |
| Formic Acid | 0.03% |
| Furfural | 0.04% |
| HMF | 0.01% |
| Vanillin | n.d. |
| 4-hydroxy benzoic acid | n.d. |
| 4-hydroxy benzaldehyde | n.d. |

n.d.: not determined
