# Supplementary material for: A novel AST2 mutation generated upon whole-genome transformation of Saccharomyces cerevisiae confers high tolerance to 5-Hydroxymethylfurfural (HMF) and other inhibitors
Source: PLoS Genet. 2021 Oct 8;17(10):e1009826. doi: 10.1371/journal.pgen.1009826 (PMC8500407; doi:10.1371/journal.pgen.1009826)
Supplement: S3 Table — (DOCX) [file pgen.1009826.s010.docx]

**S3 Table. List of primers used in this study**

| **Application** | **Forward Primer** | **Reverse Primer** | |
| --- | --- | --- | --- |
| Amplification of p77 for REG2 | CTAGAAGAAAATGAGACACAATTTCAATGTATTGAAAGTTTACGTAAAATGTGGTCGGCTGGAGATCGG | AGGAACAAGAAAGTGTGTGAGAATGACTTTGAGTAATTGCGACTCTTTGGAGCCGTTATGGCGGGCATC | |
| Amplification of p77 for SAS3 | TTAATAATGTTACATGTATATGCTTATATCCAATATATACCCATCGCCGCGTGGTCGGCTGGAGATCGG | CATTAATTAGTCTCCGTATAATTTGCAGATAATGTCATTAACAGCAAACGAGCCGTTATGGCGGGCATC | |
| Amplification of p77 for DPP1 | TAAATACGTATATTTCGTATGTCATGTGGAGTATATATTCTTTTTTATTCGTGGTCGGCTGGAGATCGG | ATCAATTGTTAAAGGCAAAGAATCAGAATTAAATCATAGCAAACGACCAAAAGCCGTTATGGCGGGCATC | |
| Amplification of p77 for GIC2 | CATTTTCTGTCCTTAGCTTAAGTTTGCAGGGGCTCGAGCTGGTTGAAAGAGTGGTCGGCTGGAGATCGG | AACACTAACAAAAGGAATACCTGACAGAATACGCTATCAAAAGAACGAATAGCCGTTATGGCGGGCATC | |
| Amplification of p77 for AST2 | GAAACCTCAATAAAAATGAGGTGAAACCTAGGGAGACAGAAACTCCCATTTGTGGTCGGCTGGAGATCGG | GGAAGTAAAGACGTAACAATCAAAAGAAAGTTAAACTAGCATAGAAAGAGCCGTTATGGCGGGCATC | |
| Amplification of p77 for IES1 | CACTCCAGCTTAAACATGGCGGTTGCTTCTTCGTCATCAATTTCCGCGCGGTGGTCGGCTGGAGATCGG | ATTAACTAAGTAAAATCGAAAGTTAAATAAAGGCGACACTCTAATTAAAGAGCCGTTATGGCGGGCATC | |
| Amplification of p77 for ASG1 | AGGTAAGAGACAAAGAAAAAGGAGCGCATATTATAATTGATAAGGGCGTTGTGGTCGGCTGGAGATCGG | ATATTATAGAATATAAATCAAAGATAACGTTTTCATTCAGAGGGGTAATTAGCCGTTATGGCGGGCATC | |
| Amplification of p77 for SYC1 | GAAAATAAAAAGGTAAGTAATGACGTTCAGCACAATGGAGTTGTTAATTTGTGGTCGGCTGGAGATCGG | GACTGAACAAGAGCGGCTCACGTAATTTGCATTATAACGTAAAATGGATTTAAGCCGTTATGGCGGGCATC | |
| Amplification of p77 for TAH18 | ATGAAGATTCTAGTCAACAGTAGGCCATCTGTGTTTCCATTAATCGCTGCGTGGTCGGCTGGAGATCGG | TTTAATTATATATCTATTTAACATTATAATGCGATAAGTCTTGTTATCGAAGCCGTTATGGCGGGCATC | |
| Amplification of p77 for *GDH3* deletion | TTGGCCCAGCTCTTTGAAGAAAGGAAAAAATGCGGAGAGGGAGCCAATGAGTGGTCGGCTGGAGATCGG | TACTACATACACAGATAGTTACGAACAAAAAGAAAATAGCGCTTACGGAGCCGTTATGGCGGGCATC |  |
| Amplification of p77 for *YGL185C* deletion | ACGGATCTGCGATGAAAAGAATAGTAGGCTTTCCAGTCGTTGCAGGAGAAGTGGTCGGCTGGAGATCGG | ATCTCATCAGAAAGGACTCAGAATTGAATATTAAGAGTTCGAAATTGTAAAGCCGTTATGGCGGGCATC |  |
| Amplification of p77 for *HXT2* deletion | AAAATTTTCCGAAATCCTTTTTCCTACGCGTTTTCTTCGGGAACTAGATAGTGGTCGGCTGGAGATCGG | TTAGTAGCCATTAGCCTTAAAAAAAATCAAAGTGCTAGTTTAAGTATAATCTCAGCCGTTATGGCGGGCATC |  |
| Amplification of p77 for *FAS2* deletion | CTATATTTCCTAAATTTTCTCTGGTCTGCAGGCCAAAAACAACAACTTACGTGGTCGGCTGGAGATCGG | AATAGAAGCGACACGTTACATATTAAAAGAGGGACTACGTAGTGCTCTCTAGCCGTTATGGCGGGCATC |  |
| Amplification of p77 for *HSP82* deletion | TTTATTCATTCGAATACCTATACGTTATATTATGTTTTGTTTATAACCTAGTGGTCGGCTGGAGATCGG | GATAGAAAATAGAGTCCTATAAACAAAAGCACAAACAAACACGCAAAGATAGCCGTTATGGCGGGCATC |  |
| Amplification of p77 for *HXK2* deletion | CGAAGAAAAGAGATTTCTTTTTCTCGCGGGTAGTTTTTCCGGTCGATCGAGTGGTCGGCTGGAGATCGG | TTAGTAGCCATTAGCCTTAAAAAAAATCAAAGTGCTAGTTTAAGTATAATCTCAGCCGTTATGGCGGGCATC |  |
| Amplification of p77 for *UTH1* deletion | AATTTACCCTCCCTTAATTTTTCAAGAAATTCCAGTATGAAATTATCCGCGTGGTCGGCTGGAGATCGG | AATGAACGGAGTAAAAAAAGTACTAGCAAAAGCTTATTTGCAATATTCAAAGCCGTTATGGCGGGCATC |  |
| Amplification of p77 for *FLR1* deletion | TAAAATTGTGTTATTATAATTCAAATTTTCATGGTAAGAACATCGGCTTGTGGTCGGCTGGAGATCGG | AAAATTCTTTGAGGTTCGCTTCTTCATTTTAAAATACGTTATGATGGTGAAGCCGTTATGGCGGGCATC |  |
| Amplification of p77 for *ERG26* deletion | CAACATACTATCTTTCGATAATCGGATCAAAAAGCTCCTAACGATTGCCAGTGGTCGGCTGGAGATCGG | TTCATATTTTTTCTTTCTTTGAAAGAATATTGACGACAAAGTATTGGATAAGCCGTTATGGCGGGCATC |  |
| Allele-specific PCR for *REG2* | Wild type: TGATAATGGCAGTGGCGGC  Putatively mutant: TGATAATGGCAGTGGCGGA | TTTAACGATAACGTTGAACAGTG |  |
| Allele-specific PCR for *SAS3* | Wild type: CTTCTTCTTCTTCTTCTTCGACG  Putatively mutant: CTTCTTCTTCTTCTTCTTCGAGA | TCGATTCTTGGAACAGGATTG |  |
| Allele-specific PCR for *DPP1* | Wild type: ATCATCAATGGGTGGGACGA  Putatively mutant: ATCATCAATGGGTGGGACGG | ACTGAATCACCGTTGATGC |  |
| Allele-specific PCR for *GIC2* | Wild type: GTTTCGTTTCCAGTATTGGTGAT  Putatively mutant: GTTTCGTTTCCAGTATTGGTGAC | GCTAGTGATCATTCGCATCC |  |
| Allele-specific PCR for *AST2* | Wild type: TGTAGGAAAATGCTTCCTTGAGAT  Putatively mutant: TGTAGGAAAATGCTTCCTTGAGAA | GTCTTTGATTCTTGGGATAACC |  |
| Allele-specific PCR for *IES1* | Wild type: ATTTTCTGACACATGCTTGTGCA  Putatively mutant: ATTTTCTGACACATGCTTGTGCG | GGAATCTTCTTCACCCATGAC |  |
| Allele-specific PCR for *ASG1* | Wild type: TTAAGATTGATAACAATTCACTGC  Putatively mutant: TTAAGATTGATAACAATTCACTGT | GCTGAGCATCTAACTGATCG |  |
| Allele-specific PCR for *SYC1* | Wild type: TTCGATCTGTCCCACTGGTA  Putatively mutant: TTCGATCTGTCCCACTGGTC | GTTCTTCTTCGGGAAAGGAAG |  |
| Allele-specific PCR for *TAH18* | Wild type: GAACATGCCAATGAAAAGTGAAA  Putatively mutant: GAACATGCCAATGAAAAGTGAAC | CATCCTCATCTGTATTGCAAGG |  |
| Amplification of *GDH3* | GTGATGACCATGATGCCTT | CTCTGGTTCGCTTGTCA |  |
| Amplification of *YGL185C* | AATATACTTACTGTTCTTGAAATTCTGT | GATGACCTGCATAAGCCTT |  |
| Amplification of *HXT2* | TAACGATAGTAATAGGCCAC | TCTCAAATCTGGTTAGAATGTA |  |
| Amplification of *FAS2* | ACGTGTGTTTAGTTTAGCCA | TGTGTAACCTTCAGTTAACAAG |  |
| Amplification of *HSP82* | CAGCCTTGGTCATACCAATAC | ATGGCTAGTGAAACTTTTGAATT |  |
| Amplification of *HXK2* | ACGGTACTTTCTCTGTTTCAG | AGATATGGGGTAGGATTACTCT |  |
| Amplification of *UTH1* | GTTGTTATTCCAGCTGCCA | CTTGTCCGGATGGGAAAT |  |
| Amplification of *FLR1* | ATAGCTTCACCATAATGACAAAG | TCTCTACAATATTTCGCATCACT |  |
| Amplification of *ERG26* | GTTTGCCCTTCCAGGTAG | TGTAGATCTTCGGTTCAATGA |  |
| gRNA cloning for *AST2* | GCAGTGAAAGATAAATGATCTTATTCCTGGAAAAATTTCAGTTTTAGAGCTAGAAATAG | CTATTTCTAGCTCTAAAACTGAAATTTTTCCAGGAATAAGATCATTTATCTTTCACTGC |  |
| gRNA cloning for *AST1* | GCAGTGAAAGATAAATGATCTATAAGAAAATGCTTCTTTAGTTTTAGAGCTAGAAATAG | CTATTTCTAGCTCTAAAACTAAAGAAGCATTTTCTTATAGATCATTTATCTTTCACTGC |  |
